# The Discovery and Development of Liraglutide and Semaglutide

**DOI:** 10.3389/fendo.2019.00155

**Published:** 2019-04-12

**Authors:** Lotte Bjerre Knudsen, Jesper Lau

**Affiliations:** ^1^Global Drug Discovery, Novo Nordisk A/S, Måløv, Denmark; ^2^Global Research Technology, Novo Nordisk A/S, Måløv, Denmark

**Keywords:** GLP-1, liraglutide, semaglutide, type 2 diabetes, obesity, albumin, once-weekly

## Abstract

The discovery of glucagon-like peptide-1 (GLP-1), an incretin hormone with important effects on glycemic control and body weight regulation, led to efforts to extend its half-life and make it therapeutically effective in people with type 2 diabetes (T2D). The development of short- and then long-acting GLP-1 receptor agonists (GLP-1RAs) followed. Our article charts the discovery and development of the long-acting GLP-1 analogs liraglutide and, subsequently, semaglutide. We examine the chemistry employed in designing liraglutide and semaglutide, the human and non-human studies used to investigate their cellular targets and pharmacological effects, and ongoing investigations into new applications and formulations of these drugs. Reversible binding to albumin was used for the systemic protraction of liraglutide and semaglutide, with optimal fatty acid and linker combinations identified to maximize albumin binding while maintaining GLP-1 receptor (GLP-1R) potency. GLP-1RAs mediate their effects via this receptor, which is expressed in the pancreas, gastrointestinal tract, heart, lungs, kidneys, and brain. GLP-1Rs in the pancreas and brain have been shown to account for the respective improvements in glycemic control and body weight that are evident with liraglutide and semaglutide. Both liraglutide and semaglutide also positively affect cardiovascular (CV) outcomes in individuals with T2D, although the precise mechanism is still being explored. Significant weight loss, through an effect to reduce energy intake, led to the approval of liraglutide (3.0 mg) for the treatment of obesity, an indication currently under investigation with semaglutide. Other ongoing investigations with semaglutide include the treatment of non-alcoholic fatty liver disease (NASH) and its use in an oral formulation for the treatment of T2D. In summary, rational design has led to the development of two long-acting GLP-1 analogs, liraglutide and semaglutide, that have made a vast contribution to the management of T2D in terms of improvements in glycemic control, body weight, blood pressure, lipids, beta-cell function, and CV outcomes. Furthermore, the development of an oral formulation for semaglutide may provide individuals with additional benefits in relation to treatment adherence. In addition to T2D, liraglutide is used in the treatment of obesity, while semaglutide is currently under investigation for use in obesity and NASH.

## Introduction

Bayliss and Starling first described the connection between the pancreas, the gut and incretin hormones in the early part of the twentieth century (1). When the incretin hormone glucagon-like peptide-1 (GLP-1) was subsequently shown to account for up to 70% of insulin secretion in response to nutrient intake (2), its potential as a therapeutic target in type 2 diabetes (T2D) was realized. However, while the insulin secretory response could be restored with pharmacological levels of native GLP-1 in patients with T2D (2, 3), a short half-life limited its therapeutic use (4).

Various approaches have since been used to extend the half-life of native GLP-1, several of which have resulted in pharmacological agents that are effective in the treatment of T2D. We describe one such approach, albumin binding, and explain how it was applied in the development of the human GLP-1 analog liraglutide once daily and, subsequently, semaglutide once weekly. The pharmacology of these two long-acting GLP-1 analogs, in terms of improving glycemic control, reducing body weight and decreasing cardiovascular (CV) risk, is also reviewed, together with some novel biology. In addition, we describe the importance of accurate target (GLP-1 receptor) tissue expression analysis.

Now an established class of agents, GLP-1-based therapies represent a significant advance in the treatment of T2D. All currently available GLP-1 receptor agonists (GLP-1RAs) are, however, injectable. Ongoing efforts to advance this class of agents include, therefore, attempts to develop an oral formulation. In the final section of this article, we describe an oral formulation of semaglutide that is currently in phase 3 clinical development for the treatment of T2D, as well as summarizing other therapeutic applications—beyond T2D—of liraglutide and semaglutide.

## Albumin Binding as a Concept for Creating Long-Acting GLP-1 Analogs

Human serum albumin (HSA) is among the most stable and abundant of plasma proteins. It comprises three homologous domains assembled into a heart-shaped protein. Approximately 10–15 g is produced by the liver daily, which—owing to its long half-life of several weeks—results in an HSA plasma concentration of 35–50 g/L (~0.6 mM) (5). The long half-life of HSA is due to its ability to pH-dependently bind the neonatal receptor (FcRn), in the same manner as the Fc domain of antibodies (6). High-affinity binding of HSA to FcRn recycles the protein back to plasma, thus protecting it from degradation during endocytosis (7). In the plasma HSA is released due to its low affinity for FcRn at a neutral pH (8). HSA also plays an important role in achieving homeostasis, by stabilizing plasma pH and denaturation conditions. Furthermore, it is an important antioxidant (9).

**Figure 1:**
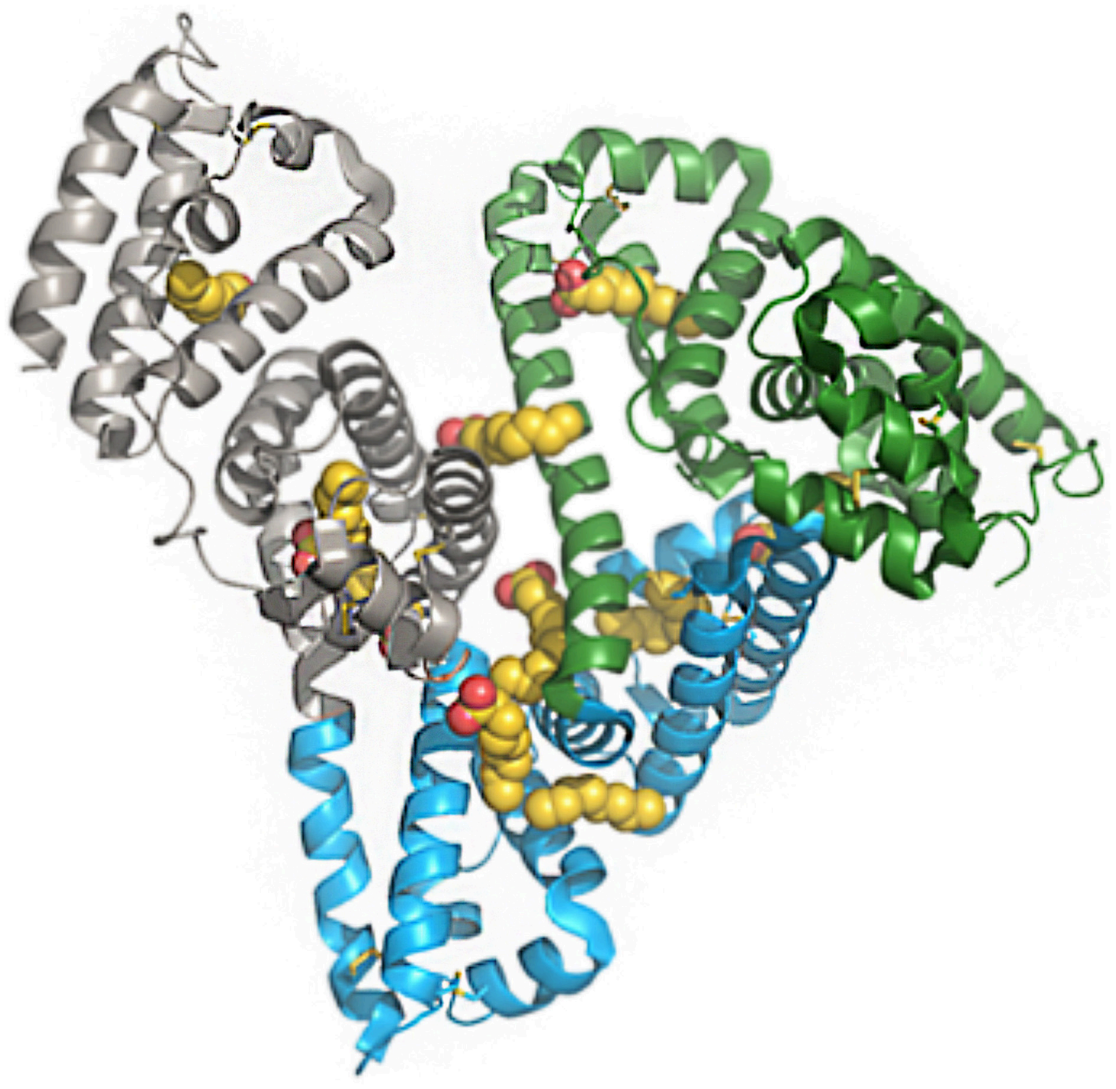
Structure of human serum albumin, representing domains and fatty-acid binding sites. Green, subdomain I; blue, subdomain II; gray, subdomain III; yellow/red, fatty acids. The PDB file has code 1E7E resolved to 2.5 Å resolution. Reprinted from Bhattacharya et al. (10). Copyright 2018, with permission from Elsevier.

A feature of HSA of particular interest is its ability to bind to a variety of components in plasma, thereby facilitating the solubility and transportation of otherwise insoluble substrates, such as fatty acids and steroids. As shown in Figure 1, multiple binding sites for fatty acids have been elucidated since the structure of albumin was published in 1992 (10–13).

Albumin also binds to a wide variety of drugs and numerous efforts investigating this have concluded that the most common binding sides, drug site 1 and 2, are located in the IIA and IIIA subdomains, respectively. Both of these sites comprise large apolar cavities with defined polar features (13). Crystallographic analysis has revealed where, and for how long, fatty acids bind to albumin. The fatty acids C10, C12, C14, C16, and C18 have been shown to bind to seven sites on albumin (13). The combination of high plasma concentration, several binding sites, and a long half-life gives albumin an extensive capacity to transport fatty acids in the systemic compartments (13–15). Several approaches for using albumin as a carrier in drug discovery have been investigated. Various ligands have been identified that bind to albumin, including small molecules, peptides, and proteins. Since this review is about peptide ligands, the focus is on peptide- and protein-based pharmaceuticals, and the many examples of small molecules that bind to albumin are not discussed.

The first high-affinity, *de novo*, peptide-based ligands were discovered using phage display (16). A unique binding epitope on albumin gave a 1:1 binding stoichiometry for a cyclic peptide with the core sequence DICLPRWGCLW, but showed species-specific differences in binding affinity. This albumin-binding peptide was fused to fab fragments, which increased the half-life significantly in mice (37-fold) and rabbits (26-fold) (17). Domain antibodies and small protein scaffold have also been shown to bind to albumin, and used to extend the half-life of substrates otherwise rapidly cleared by renal filtration (18, 19).

The concept of using fatty acids as albumin tags to prolong the duration of action of peptides and proteins has been extensively investigated by Novo Nordisk and others. In 1998, the first description of this approach was published featuring His7, Arg26 GLP-1(7-37) derivatized with a C8 fatty acid (20). The compound did not appear to have strong albumin binding and was subject to self-association and physical instability, limiting its pharmaceutical use (21).

Insulin detemir was the first clinically approved protein modified by a fatty acid. Myristic acid, attached to the B29 lysine of desB30 insulin, increased the duration of action of insulin detemir to an extent that made it applicable for once-daily (OD) injection (22, 23). The underlying mechanism for this increase is primarily subcutaneous deposition after injection, with only a small increase in intravenous half-life due to albumin binding (22, 24, 25).

Insulin degludec is the latest generation of albumin-binding insulin to be based on fatty-acid derivatization. Hexadecandionyl was attached to desB30 human insulin via a L-γ-glutamic acid spacer. Subcutaneous deposition was also an important part of the mechanism of protraction here, facilitated by the fatty acid in insulin degludec leading to self-assembly into multi-hexamers, but insulin degludec also has a longer intravenous half-life than insulin detemir (26).

## The Discovery of Liraglutide

GLP-1 is a 30 amino acid peptide hormone with a short half-life (1.5 min following intravenous dosing and 1.5 h following subcutaneous dosing in humans) (4). These properties have posed challenges to the pharmaceutical use of GLP-1, where a constantly high and stable plasma level is required (27). Furthermore, native GLP-1 is subject to fibrillation, as best described for glucagon (28–31).

In attempts to prolong the half-life of GLP-1 and develop a suitable compound for therapeutic use, some pharmaceutical companies (32) chose to optimize the exendin-4 peptide (a naturally occurring peptide obtained from Heloderma lizard venom, with 53% homology to human GLP-1), which leads to an intravenous half-life of ~30 min (33–35). We used human GLP-1 as the starting point for our drug discovery program. As it was very important to increase the intravenous half-life of GLP-1 beyond that which could be obtained with dipeptidyl peptidase IV (DPP-IV) stabilization (36), fatty-acid derivatization was employed. The aim was to prepare analogs that could bind to albumin in a reversible manner and, thereby, protect the peptide from both DPP-IV degradation and renal filtration.

Initially, a systematic alanine (Ala) scan of the entire GLP-1 sequence was conducted to understand the role of each amino acid in the peptide (37). The Ala scan revealed the N-terminal (position 7, 8, 9, 10, 12, 13, and 15), as well as specific amino acids toward the C-terminal (position 28 and 29), to be crucial for the activity of GLP-1. Another important observation regarding the N-terminal was that, while the N-terminal of GLP-1 is susceptible to DPP-IV degradation, the N-terminal of exendin-4 is resistant to DPP-IV degradation. This difference is due to a single amino acid at the 2nd position in the N-terminal, where GLP-1 has an alanine (Ala8) while exendin-4 has a glycine (Gly2). An early study showed that DPP-IV lability of GLP-1 could be reduced by introducing alternative amino acids at position 8 (36). However, while introducing Gly at position 8 reduced the DPP-IV lability of GLP-1, its affinity for the GLP-1 receptor (GLP-1R) was also significantly reduced. The only Ala substitution that resulted in both DPP-IV stability and high GLP-1R affinity was the introduction of aminoisobutyric acid (Aib) at position 8 (36).

Studies of the structure of class C G-protein coupled receptors (GPCRs) have provided insight on the specific interactions between peptides and receptors (38–40), and the findings from such studies are aligned with the results from the original Ala scan. It is, thus, concluded that the C-terminal of GLP-1 binds to the extracellular domain, whereas the N-terminal binds to the transmembrane domain of the receptor. Previous solution-phase studies and computational models of GLP-1 and exendin-4 indicated that the N-terminal had a type II beta turn, but more recent results concluded that the secondary structure of peptides, when bound to class B GPCRs of which the GLP-1R is one, is helical all through the sequence (41).

Building on the findings from the Ala scan, a series of GLP-1 analogs were derivatized with various fatty acids to investigate the effect of the attachment of fatty-acid side chains, as shown in Table 1 (42). A gamma glutamate (γGlu) linker was introduced to compensate for the loss of the acidic group used for amide linkage. An initial conclusion was that fatty acids equal to, or longer than, 12 carbons atoms resulted in half-lives of more than 9 h following subcutaneous dosing compared with 1.5 h for native GLP-1. The position of derivatization was also investigated and it was concluded that receptor potency was substantially diminished when the fatty acid was near the N-terminal. This is shown in Table 1 (42). These findings were consistent with the structure activity findings reported in the Ala scan (37). Thus, positions 7, 10, 12, 13, and 15 in the N-terminal, as well as positions 28 and 29 in the C-terminal, of GLP-1 were unsuitable for substitution with a fatty-acid derivatized lysine. While there were several possibilities for derivatization toward the C-terminal, as there is already a lysine in position 26 available for chemical derivatization, this was a preferred position for the attachment of a fatty acid. An additional benefit was that the only other lysine, at position 34, could be substituted with arginine resulting in a GLP-1 analog that could be used in a semi-recombinant process, where the peptide backbone was produced recombinantly and the fatty acid attached afterwards by a simple chemical reaction (42).

**Table 1:** Compounds and their potency measured using the cloned human GLP-1 receptor expressed in baby hamster kidney cells.

**Compound**	**Parent peptide**	**Acyl site**	**Acyl substituent**	**Potency (EC_**50**_, pM)**
1	GLP-1(7-37)		None	55 ± 19
2	K^8^R^26, 34^-GLP-1(7-37)	K^8^	γ-Glu-C16	1260 ± 210
3	K^18^R^26, 34^-GLP-1(7-37)	K^18^	γ-Glu-C16	35.2 ± 6.2
4	K^23^R^26, 34^-GLP-1(7-37)	K^23^	γ-Glu-C16	30.1 ± 3.3
5	R^34^-GLP-1(7-37)	K^26^	γ-Glu-C16	61.0 ± 7.1
6	K^27^R^26, 34^-GLP-1(7-37)	K^27^	γ-Glu-C16	36.3 ± 0.3
7	R^26^-GLP-1(7-37)	K^34^	γ-Glu-C16	121 ± 26
8	K^36^R^26, 34^-GLP-1(7-36)	K^36^	γ-Glu-C16	36.4 ± 2.1
9	R^26, 34^-GLP-1(7-38)	K^38^	γ-Glu-C16	53.0 ± 2.8
10	GLP-1(7-37)	K^26, 34^	bis-C16-diacid	7,000 ± 7
11	GLP-1(7-37)	K^26, 34^	bis-γ-Glu-C16	16,700 ± 3,700
12	GLP-1(7-37)	K^26, 34^	bis-γ-Glu-C14	3,050 ± 350
13	GLP-1(7-37)	K^26, 34^	bis-C12-diacid	177 ± 52
14	R^34^-GLP-1(7-37)	K^26^	C16-diacid	154 ± 66
15	R^34^-GLP-1(7-37)	K^26^	C14-diacid	72 ± 0.7
16	R^34^-GLP-1(7-37)	K^26^	γ-Glu-C18	194 ± 24
17	R^34^-GLP-1(7-37)	K^26^	γ-Glu-C14	22.0 ± 7.1
18	R^34^-GLP-1(7-37)	K^26^	γ-Glu-C12	27.3 ± 8.4
19	desamino-H^7^R^34^-GLP-1(7-37)	K^26^	γ-Glu-C16	687 ± 129
20	R^34^-GLP-1(7-37)	K^26^	GABA-C16	84.4 ± 22.1
21	R^34^-GLP-1(7-37)	K^26^	β-Ala-C16	113 ± 3
22	R^34^-GLP-1(7-37)	K^26^	Iso-Nip-C16	410 ± 120
23	desamino-H^7^R^26^-GLP-1(7-37)	K^34^	γ-Glu-C16	2360 ± 370
24	desamino-H^7^R^26^-GLP-1(7-37)	K^34^	C8	236 ± 66
25	desamino-H^7^R^26^-GLP-1(7-37)	K^34^	γ-Glu-C8	169 ± 1
26	K^36^R^26, 34^-GLP-1(7-36)	K^36^	C20-diacid	210 ± 14
27	K^36^R^26, 34^-GLP-1(7-36)	K^36^	C16-diacid	7.89 ± 1.21
28	K^36^R^26, 34^-GLP-1(7-36)	K^36^	γ-Glu-C18	116 ± 3
29	R^26, 34^-GLP-1(7-38)	K^38^	C16-diacid	5.60 ± 3.5
30	R^26, 34^-GLP-1(7-38)	K^38^	C12-diacid	4.19 ± 0.98
31	R^26, 34^-GLP-1(7-38)	K^38^	γ-Glu-C18	115 ± 21
32	R^26, 34^-GLP-1(7-38)	K^38^	γ-Glu-C14	54 ± 1
33	G^8^R^26, 34^-GLP-1(7-38)	K^38^	γ-Glu-C16	328 ± 14
34	E^37^R^26, 34^-GLP-1(7-38)	K^38^	γ-Glu-C16	27.2 ± 0.1
35	E^37^G^8^R^26, 34^-GLP-1(7-38)	K^38^	γ-Glu-C16	135 ± 7
36	E^37^G^8^R^26, 34^-GLP-1(7-38)	K^38^	γ-Glu-C18	213 ± 30

*Abbreviations used for acyl groups in lysine N^ϵ^-acylated peptides: γ-Glu-C8, γ-L-glutamoyl(N^α^-octanoyl); γ-Glu-C14, γ-Lglutamoyl(N^α^-tetradecanoyl); γ-Glu-C16, γ-L-glutamoyl(N^α^-hexadecanoyl); γ-Glu-C18, γ-L-glutamoyl(N^α^-octadecanoyl); C8, octanoyl; C12-diacid, ω-carboxyundecanoyl; C16-diacid, ω-carboxypentadecanoyl; C20-diacid, ω-carboxynonadecanoyl; GABA-C16, γ-aminobutyroyl(N^γ^-hexadecanoyl); Iso-Nip-C16, 1-(hexadecanoyl)piperidyl-4-carboxy. Data are given as mean ± standard deviation of two individual experiments with triplicate samples. EC_50_, effective concentration. Source: Knudsen et al. (42)*.

Further investigation revealed that the use of fatty di-acids longer than 14 carbons attached via γGlu resulted in loss of activity, while the use of mono-acids of up to 16 carbons (palmitate) retained activity, as shown in Table 1. Furthermore, the attachment of two fatty acids had a negative effect on receptor potency. The use of other chemical spacers was also explored, with the aim of substituting the optically active γGlu with a simple, non-chiral spacer (42). It was concluded that the use of palmitate with a γGlu linker was the optimal combination to obtain an appropriate *in vivo* protraction without compromising receptor potency.

Based on these initial studies and comprehensive characterization, liraglutide was selected as having the best properties, combining high receptor potency with pharmacokinetics (PK) that are optimum for OD dosing (43). A key property of liraglutide is its partial protection from rapid DPP-IV degradation, despite the His-Ala N-terminal being unchanged (44). This protection may be due to the reversible binding to albumin, or direct steric hindrance. Further studies revealed that this peptide, in addition to having an extended elimination half-life, has a delayed subcutaneous absorption (45). Biophysical investigations showed that the drug formulation of liraglutide contains a self-assembled hepta-peptide that may partially explain its delayed subcutaneous absorption (45).

Following the selection of liraglutide as the first GLP-1-based analog suitable for OD dosing, further analysis of its structural activity was published (46). It was concluded that there was a good correlation between PK and the length of the fatty acid when using linear mono-acids. An additional conclusion was that the chemical spacer between the fatty acid and the peptide might be important for receptor potency, although its presence had little impact on the PK in pigs (46).

## The Discovery of Semaglutide

Successful clinical trials with exenatide and liraglutide led to an increased interest in GLP-1-based therapies. As daily injections are a barrier for some patients with T2D, there was focus on improving convenience, ideally with an effective GLP-1 analog that could be administered once weekly.

Several technologies have been explored to discover and develop a GLP-1RA applicable for once-weekly (OW) dosing. Sustained release was one of the first approaches to be assessed in clinical trials, and led to approval of the encapsulated formulation of exenatide: exenatide extended release (ER) (47). The first human-based GLP-1RA to be evaluated in clinical trials for OW dosing was taspoglutide (BIM-51077, Aib8,35 GLP-1 [7-36] amide, Roche). The Aib8 protected taspoglutide from DPP-IV degradation (48). Although a zinc chloride-based formulation of taspoglutide, facilitating subcutaneous precipitation, showed promising results, phase 3 trials were completed, but a submission for approval was discontinued, due to a number of cases of anaphylactic shock (49, 50). Other approaches have entailed limiting the renal clearance of GLP-1- or exendin-based compounds by covalent fusion of the peptide to a large, stable plasma protein like albumin (albiglutide) (51) or the Fc domain of IgG (dulaglutide) (52).

At Novo Nordisk the idea was to build on reversible binding to albumin as a solution for the systemic protraction of GLP-1 analogs. The main challenge identified in earlier studies was that strong binding to albumin had a negative impact on the potency of compounds for the GLP-1R, due to competition between binding to albumin and binding to the receptor (42, 46). The theory was that only the free fraction in the plasma that was not bound to albumin would be available to activate the GLP-1R. Therefore, the stronger the affinity to albumin the smaller the free and active circulating fraction of the GLP-1 peptide. This phenomenon had previously been observed with liraglutide analogs, where there was a clear trend for longer fatty acids, with improved affinity for albumin, to be associated with diminished potency for the GLP-1R (53). These findings were further supported by studies with albiglutide, where the potency of the GLP-1RA binding covalently to albumin necessitated a high dose to obtain clinically relevant efficacy (54). Albiglutide has a tandem repeat of Gly8 GLP-1(7-36). The purpose of the tandem repeat is likely to improve affinity for the GLP-1R, by creating a longer distance between albumin and the distal GLP-1 peptide (55). An amino acid substitution (Ala8 to Gly8) protects albiglutide from DPP-IV degradation at the N-terminal. Finally, the tandem repeat is fused to the N-terminal of HSA to extend the half-life of albiglutide. However, while the half-life was extended to 6–8 days, making it suitable for OW dosing (56), the potency of albiglutide was significantly reduced (GLP-1R affinity of albiglutide is 20 nM compared with 0.02 nM for exenatide) (55), most likely due to a combination of the Gly8 modification and the covalent attachment to HSA.

**Figure 2:**
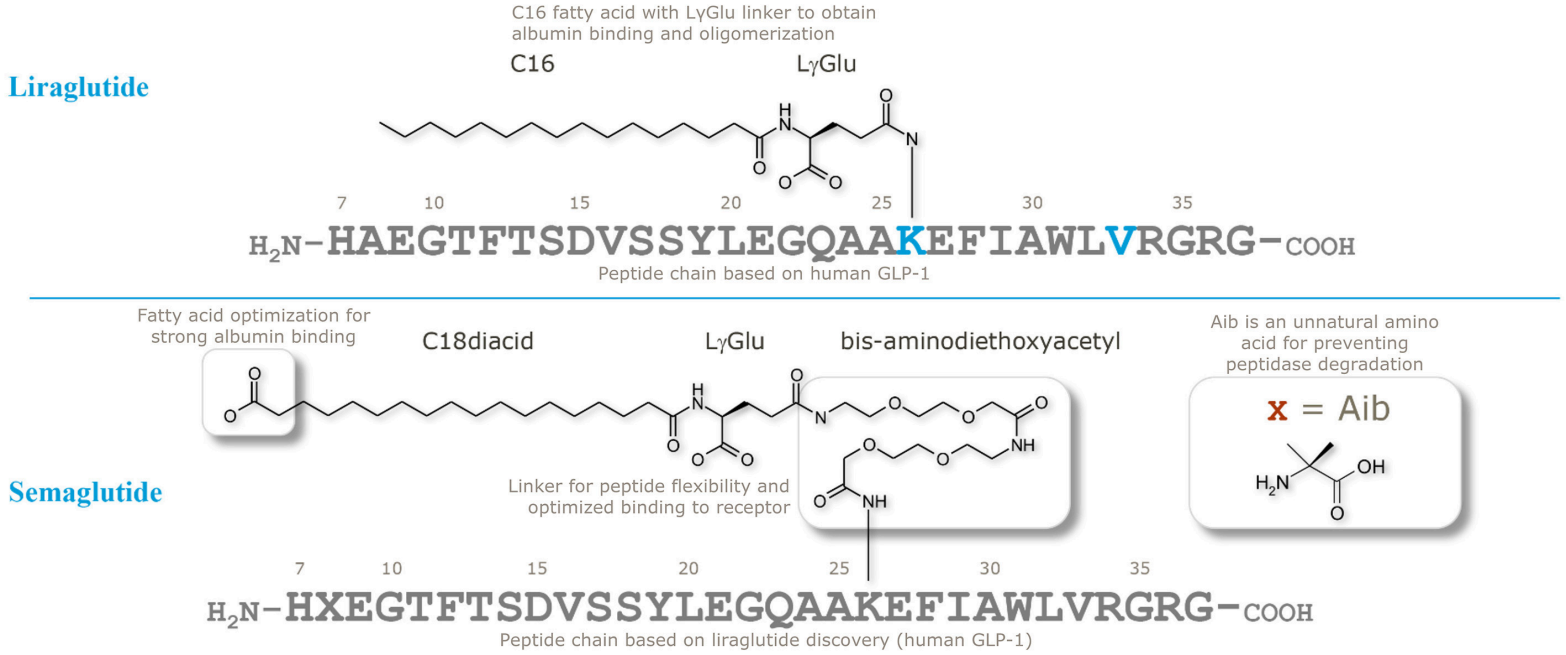
Chemical structures of liraglutide and semaglutide. Adapted with permission from Lau et al. (53). Copyright 2018, American Chemical Society.

When designing semaglutide, a realistic concern was if it would be possible to stabilize a GLP-1 analog against systemic clearance to achieve plasma levels sufficient to control blood glucose following OW dosing. Initial findings with liraglutide, using both *in vitro* and *in vivo* (animal models) GLP-1R assays, had shown it was challenging to achieve an optimal balance between a long plasma half-life and sufficiently high GLP-1R affinity in the presence of albumin (46); this is evidenced by the relatively higher clinical dose of liraglutide (up to 1.8 mg once daily) vs. exenatide (2^*^20 μg). In the comprehensive research program to solve this challenge, the strategy was to keep the peptide as similar to liraglutide and endogenous GLP-1 as possible, to avoid unnecessary risks in terms of immunogenicity. Thus, as illustrated in Figure 2, the peptide backbone was modified at position 8 only, where Ala was substituted with Aib, previously shown to be resistant to DPP-IV cleavage and have high GLP-1R affinity (36). The second part of the strategy was to find the optimal combination of a fatty acid with high albumin affinity, attached to GLP-1 via a water compatible chemical linker, to ensure that the derivatized peptide had high GLP-1R potency in the presence of albumin. Several fatty acids and linkers were systematically assessed (53). Based on linker studies, where palmitate was used as an albumin tag, it was observed that the introduction of “OEG” linkers resulted in both high receptor potency and albumin affinity. To increase albumin affinity, beyond that obtained with palmitate, the length and type of fatty acid was explored. Increasing the length of fatty acids from C16 (palmitate) to C18 or C20 did not result in the desired properties. Fatty di-acids that had a proximal fatty acid for amide connectivity to the linker, as well as a distal fatty acid, were shown to be the solution to obtaining high albumin binding and GLP-1R potency.

A systematic test of derivatives from C12 to C20 fatty acids showed that a C18 di-acid together with a γGlu-2xOEG linker resulted in the highest albumin affinity combined with GLP-1R potency. Structure–activity relations of the derivatives clearly demonstrated that the length of fatty di-acid was important, and that the C18 di-acid used in semaglutide was the optimal choice. There was a clear trend for increasing GLP-1R potency with increasing carbon atoms in di-acids from C12 to C18, but this trend was reversed when longer (>C18) di-acids were used. In a receptor-binding assay the C18 di-acid was also shown to have the highest affinity shift when albumin was added, indicating a strong albumin affinity (53).

**Figure 3:**
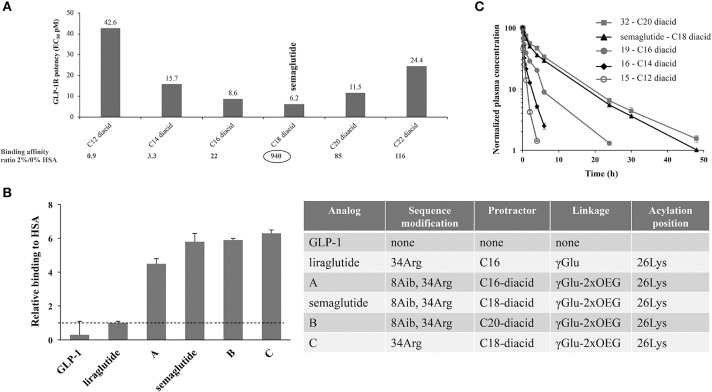
Albumin affinity of fatty-acid derivatized peptides vs. native GLP-1. **(A)** Derivatives of Aib8, Arg34 GLP-1(7-37) with C12 to C22 di-acids and attached to Lys26 using γGlu- and OEG-based linkers. GLP-1R binding at 0 and 2% HSA using baby BHK cells expressing the human GLP-1R, and *in vitro* potency measured in BHK cells that express both the human GLP-1R and a luciferase reporter system; **(B)** HSA binding of GLP-1 derivatives, assessed using analytical ultracentrifugation; **(C)** *In vivo* protraction in rats following intravenous administration of 32 (5.5 nmol/kg), semaglutide (4.2 nmol/kg), 19 (3.3 nmol/kg), 16 (5.5 nmol/kg), and 15 (5.3 nmol/kg). BHK, baby hamster kidney; EC_50_, effective concentration; GLP-1, glucagon-like peptide-1; GLP-1R, glucagon-like peptide-1 receptor; HSA, human serum albumin. Adapted with permission from Lau et al. (53). Copyright 2018, American Chemical Society.

Direct measurement of albumin affinity is challenging with amphiphilic and lipophilic peptide derivatives. It has, however, been possible to measure relative albumin affinities using analytical ultracentrifugation. As seen in Figure 3, the fatty-acid derivatized peptides all had a higher albumin affinity than native GLP-1 (53). It was also clear that derivatives comprising a di-acid had higher albumin affinity compared with liraglutide, and that the length of the di-acid was important (53).

**Figure 4:**
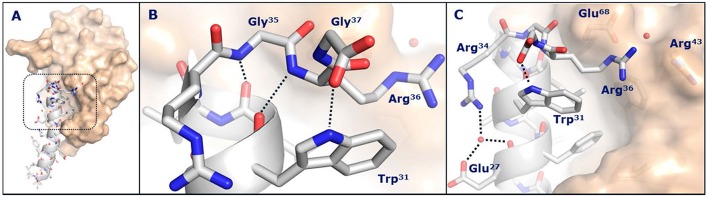
Crystal structure of the semaglutide peptide backbone (gray) in complex with the GLP-1 receptor extracellular domain (golden surface). Individual residues are shown as sticks, with nitrogen and oxygen atoms colored blue and red, respectively. **(A)** Overall structure with boxed area magnified in **(B,C)**. **(B)** The structure of the C-terminus of the semaglutide peptide backbone. Hydrogen bond interactions are illustrated as dotted lines. **(C)** Arg36 closes the hydrophobic ligand-receptor interface by aligning with Trp31 and Glu68. A water molecule is coordinated by Glu27 and Arg34. With permission from Lau et al. (53). Copyright 2018, American Chemical Society.

While it was not possible to obtain a crystal structure of semaglutide, crystallization of the unacylated semaglutide peptide backbone in complex with the GLP-1R showed that the overall structure was identical to that of native GLP-1(7-37). In both structures, Lys26 interacted with Glu128 of the GLP-1R, despite previous receptor mutagenesis studies suggesting that this interaction was not important and that acylation of Lys26 had only a minor impact on receptor affinity. The native Lys34 of GLP-1(7-37)-OH appeared highly flexible in complex with the GLP-1R extracellular domain, whereas Arg34 of semaglutide adopted a more defined conformation oriented toward Glu27, as depicted in Figure 4. In this structure, a water molecule was coordinated by the guanidine group of Arg34, the backbone carbonyl and carboxyl group of Glu27. The water molecule appeared to mediate an electrostatic interaction between Arg34 and Glu27. Arg34 was introduced into liraglutide to enable site-specific acylation of Lys26 and had no apparent effect on binding to the GLP-1R (53).

*In vivo* kinetics and dynamics were also key considerations during the screening program for the selection of OW semaglutide. The early program included PK studies in rats and mini pigs, as well as efficacy in db/db mice, to select derivatives that combined a long PK profile with high *in vivo* GLP-1R potency (53). For the sub-series of di-acid derivatized peptides that were selected based on *in vitro* assays, *in vivo* properties were shown to be largely aligned. In Figure 3C, we see that, in rats, the half-life of the derivatives increased with the length of the fatty acids, from 1.2 h for C12 di-acid to around 7 h for semaglutide, and even longer for the C20 di-acid. As PK data in rats are not always reflective of those in humans, the most promising derivatives were also tested in mini pigs; these studies demonstrated a longer half-life than that observed in rats (53). Another difference in the findings in mini pigs vs. rats related to the half-life of the C20 di-acid derivative; while this derivative had a longer half-life than C18 (semaglutide) in rats, the apparent half-life in mini pigs was similar to semaglutide at about 75 h following subcutaneous dosing and 55 h following intravenous dosing (53).

The efficacy of semaglutide was initially demonstrated in db/db mice. A dose-response study of the most promising derivatives confirmed a high potency and duration of action with an ED50 below 2 nmol/kg (calculated as the delta area under the curve [AUC] for blood glucose 48 h following subcutaneous dosing) (53). Based on this study, semaglutide was selected to progress to more comprehensive studies and, subsequently, as the compound to evaluate in clinical trials (53).

The remainder of this review describes the pharmacology of liraglutide and semaglutide. As an inherently important part of understanding the pharmacology of any drug involves discerning its cellular targets, we begin with an overview of the most important receptor populations and the organs where GLP-1R expression is questionable from a drug mechanism of action point of view. In addition, we provide some pharmacological context for the organs where GLP-1R expression is most relevant. Subsequently, we explore the pharmacology of liraglutide and semaglutide and how this has informed our knowledge of the mechanism of action of these drugs. The focus in this review is on studies conducted by the authors, providing an overview and placing them in context of the broader literature. The article concludes with an overview of ongoing investigations with these novel analogs, including trials that indicate the feasibility of providing an oral formulation, which would represent a remarkable advance in what is currently an injectable drug class.

## GLP-1R Expression (Or Not)

The first descriptions of GLP-1(7-36) amide binding to a putative receptor in the brain (57) and pancreas (58) were published in 1987 and 1988, respectively, and activation of such receptors was shown to stimulate the adenylate cyclase pathway. Subsequently, the receptor was cloned from rats in 1992 (59) and humans in 1993 (60) and found to be a GPCR of the then newly identified sub-family that is today known as the secretin (named after the first identified member) or B class (61).

The B class of GPCRs is a relatively small group of receptors but, nevertheless, one that is important in metabolic diseases such as diabetes and obesity (62). In addition to GLP-1, this class of GPCRs includes receptors for glucagon and GIP, which are intrinsically linked to the pathophysiology or various treatment aspects of diabetes (63–65), and GLP-2 receptors that have been identified as the basis for a new treatment for short bowel syndrome (66). Both the GLP-1R and peptide are well-preserved across mammalian species, indicating their physiological importance (67, 68). While both GLP-1 and glucagon show high specificity for their receptors, glucagon also has a low affinity for the GLP-1R; however, GLP-1 does not have a measurable affinity for the glucagon receptor (69). It is the N-terminal extracellular domains of the receptors that determine specificity for peptide ligands, driven by the divergent residues in the C-terminal of the peptides (70). The first published crystal structure of the N-terminal extracellular domain of the GLP-1R was in 2003 (71), but several high-resolution GLP-1 and glucagon receptor structures have now been published (38), and these have enabled understanding of the structural parts of the binding domain down to the 3.4 Å detail level (41, 72). Knowledge about the structure of the receptor and ligand binding has been key to enabling the design of specific antibodies against the human receptor (73).

Without a complete and accurate overview of target expression at the cellular level, it is difficult to fully understand the often complex intracellular signaling and the resulting metabolic effects. While much has been published on cellular expression of GPCRs, including the GLP-1R, when evaluating the validity of such data it is key to consider the methodologies applied [e.g., immunohistochemistry (IHC), *in situ* hybridization (ISH), polymerase chain reaction (PCR), *in situ* ligand binding (ISBL)]. ISLB has high specificity and sensitivity, but provides limited cellular resolution. When used correctly ISLB is, however, a good tool to use in preliminary studies for characterizing organ expression. To obtain higher cellular resolution IHC is often used. Non-specific staining has, however, long been known to be an issue with this technique (74–76). This is because antibodies against GPCRs have a notorious lack of specificity (77–80), and antibodies for the GLP-1R are no exception (81). ISH provides high cellular resolution, especially since the development of the RNAscope protocol (82) that has increased sensitivity and specificity, but this technique only measures mRNA and not receptor protein expression. PCR is another valuable method to assess receptor expression, although it does not typically provide cellular resolution, unless laser capture microdissection is also applied; this combination of techniques may give refined spatial resolution (83, 84). As each method has its limitations, at least two carefully controlled approaches should be used to confirm a new cellular localization of the receptor. Furthermore, for effects in the brain, correct determination of neuronal subtypes, and soma vs. projection expression, is equally important.

Here, we outline the most important GLP-1R populations and also summarize those organs and cellular localizations where expression might be questionable.

Non-human primates are a good source of high-quality phylogenetic tissue, with a high translational value because of their phylogenetic similarity to humans. In the absence of high-quality human tissue, we have often used non-human primate tissue. There is close interspecies homology between the GLP-1Rs of different mammals, with the sequence of the GLP-1R in humans and rats and humans and monkeys being 90 and 99% homologous, respectively (67). In human and non-human primates GLP-1R expression in peripheral organs and the brain is generally well-characterized (73, 85–87). There are a few examples of species-specific expression, which are described in more detail below. One of the most notable species differences relates to GLP-1R expression in the thyroid; while in rats it is highly expressed in the C-cells of this organ, there appears to be little detectable expression in non-human primate and human C-cells (85, 88). GLP-1R expression has also been shown to be higher in rodent vs. human lungs (87).

### Pancreas

The pancreas is the main target organ for the action of GLP-1RAs in diabetes treatment. Functional effects in the pancreas include the glucose-dependent release of insulin, as well as an up-regulation of the biosynthesis of insulin, glucokinase, and glucose transporters. GLP-1RAs also induce glucose-dependent lowering of glucagon secretion that, in turn, lowers hepatic glucose output. In the pancreas, GLP-1Rs are predominantly localized on insulin-producing beta-cells, with a markedly weaker expression on acinar cells of the exocrine pancreas (73). Pancreatic ductal epithelial cells do not appear to express GLP-1Rs (73, 85). As the pancreas of human and non-human primates differs from that of rodents (e.g., alfa-cells in rodent islets line the mantle, whereas in primates they are more interspersed) (89, 90), conclusions based on rodent expression studies should be made with caution.

**Figure 5:**
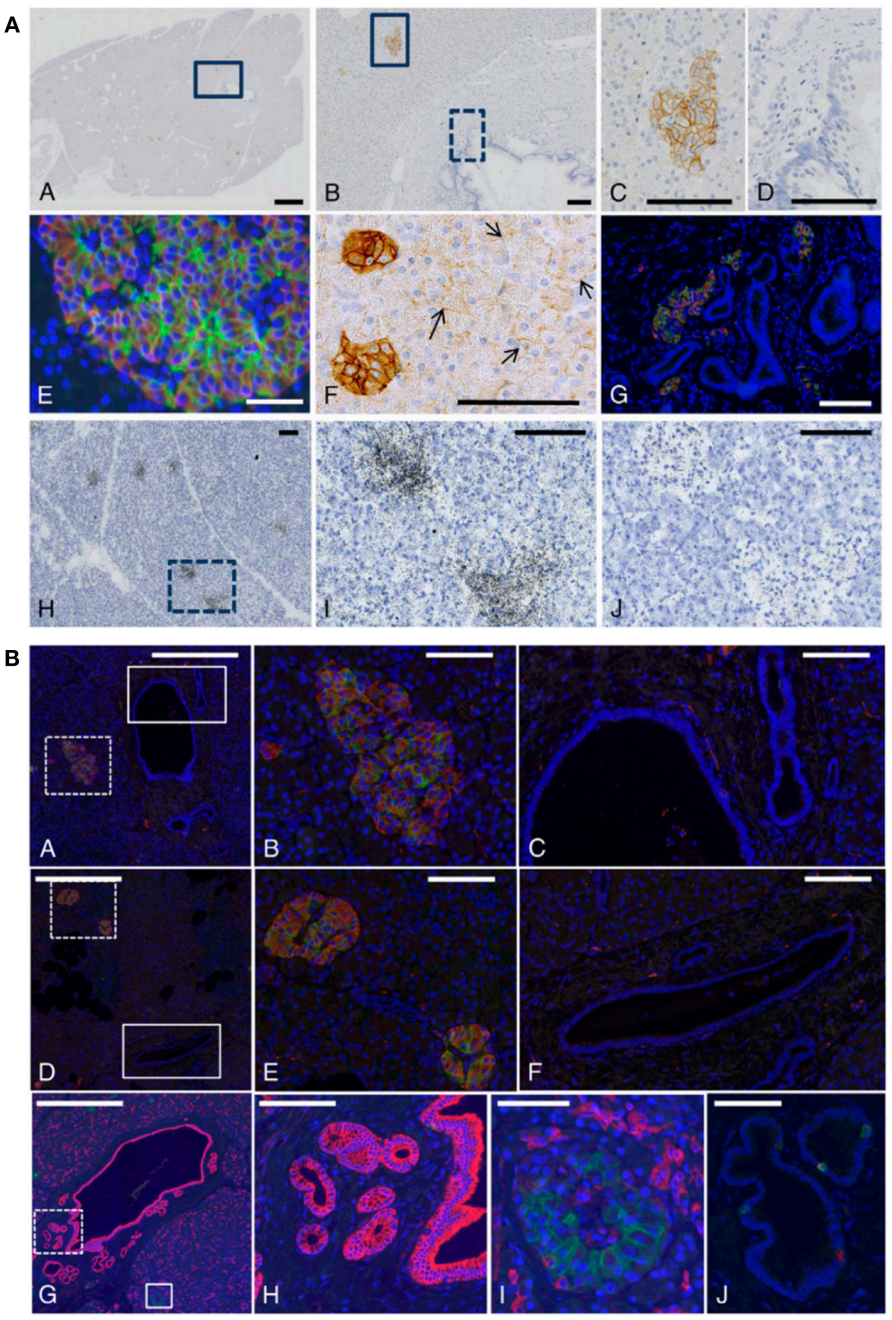
GLP-1 receptor immunoreactivity using monoclonal antibody 3F52 on monkey and human pancreas. **(A)** GLP-1R IHC with antibody MAb 3F52 on paraffin-embedded monkey pancreas (A–G) and ISLB with ^125^I-GLP-1 on a frozen section from monkey pancreas (H–J). (B,C) Magnifications of solid-line boxed areas in (A,B), respectively. (D) High magnification of the dashed-line boxed area in (B). (I) High magnification of the boxed area in (H). (A–D,F) GLP-1R immunoreactivity using a DAB IHC protocol. (E,G) Images from a double-labeling GLP-1R insulin IHC fluorescence protocol. In (E), double labeling for GLP-1R (green) and insulin (red) shows complete co-localization, demonstrating that all GLP-1R–positive cells are beta-cells. The ductal epithelium is negative for GLP-1R (D). In (F), weakly GLP-1R–immunopositive acinar cells (arrows) can be seen adjacent to strongly staining islets (left). (G) High-magnification image of the main duct area of a cynomolgus monkey pancreas double-immunostained for GLP-1R (green) and insulin (red). (J) Image from the section adjacent to (I) and incubated with ^125^I-GLP-1 plus an excess of unlabeled GLP-1. Scale bars correspond to 1 mm (A) and 100 μm (B–J). **(B)** A normal (A–C,G–J) and a diabetic (D–F) human pancreas double labeled for GLP-1R (green) and insulin (red). (B,E) High magnification images of the dashed-line boxes in (A,D), respectively. (C,F) High-magnification images of the solid-line boxes in (A,D), respectively. Note the complete co-localization of signals for GLP-1R and insulin in islets in (A,B,D,E) and absence of staining of ductal epithelium in (C,F). G-I, GLP-1R (green)/cytokeratin-19 (red) double staining in sample of normal human pancreas containing part of the main duct with PDGs. (G) Low-magnification overview. H, High magnification of area (dashed-line box in G) containing PDGs and showing no GLP-1R immunoreactivity. I, High magnification of area (solid line box in G) containing an islet with GLP-1R immunoreactive beta-cells. (J) Two GLP-1R/insulin-positive cells are located within the ductal epithelium. Scale bars correspond to 0.5 mm (A,D,G), 100 μm (B,C,E,F,H,J), and 50 μm (I). DAB, diaminobenzidine; GLP-1R, glucagon-like peptide 1 receptor; IHC, immunohistochemistry; ISBL, *in situ* ligand binding; MAb, monoclonal antibody; PDGs, pancreatic duct glands. Reproduced with permission from Pyke et al. (73). By permission of Oxford University Press on behalf of the Endocrine Society, available at: https://academic.oup.com/endo/article/155/4/1280/2423090?searchresult=1. This figure is not included under the CC-BY license of this publication. For permissions, please contact journals.permissions@oup.com.

In non-human primate tissue there was strong immunoreactivity for the GLP-1R in islet cells and predominant membrane expression was documented, as expected for a GPCR. Figure 5 outlines our results with non-human primate and human pancreatic sections (73). Beta-cells mainly accounted for the immunoreactivity, although GLP-1R expression in other rare endocrine cells cannot be fully excluded. Acinar cells showed variable, and always markedly weaker, GLP-1R immunoreactivity but were, in general, positive. This was in contrast to normal ductal epithelial cells, which were never positive (73). These findings were validated by ISLB.

Double-labeling fluorescence IHC of pancreata from healthy humans and those with diabetes showed complete co-localization of the GLP-1R and insulin in all islets and islet-like samples (73). Occasionally, GLP-1R/insulin-positive cells were found in aberrant foci; most likely representing neodifferentiation of islets from pancreatic exocrine duct epithelium or ductulo-insular complexes (73).

Specific expression of the GLP-1R in beta-cells also leads to a high expression in insulinomas (91), indeed, labeled GLP-1R ligands have been suggested as an imaging tool for detection of such tumors (92).

Discussion remains as to whether GLP-1R is expressed on glucagon-producing alfa-cells and on somatostatin-producing delta-cells, and, in turn, how GLP-1 lowers glucagon secretion. Mechanisms hypothesized to account for the effect of GLP-1 on glucagon secretion include: an indirect effect via the stimulation of somatostatin release from delta-cells or a direct effect on alfa-cells (93, 94). An additional hypothesis is that direct beta-cell to alfa-cell communication could be responsible for the glucagonostatic effect of GLP-1. This may be plausible given the interspersed heterologous contact between alfa- and beta-cells in the pancreas of primates (89, 90), and the possibility that beta-cells may have cytoplasmic extensions that span alfa-cells (89).

### Gastrointestinal Tract

Most GLP-1 is produced in the gastrointestinal (GI) tract and GLP-1R expression has classically been ascribed to myenteric plexus neurons throughout the gut (87). It is via these neurons that GLP-1 exerts its important physiological effect of regulating GI motility (95). With pharmacological administration of GLP-1, this effect is, however, subject to rapid but incomplete desensitization (95, 96). These neurons may also regulate intestinal growth through crypt fission, a novel effect of GLP-1 that has recently been identified (97). While GLP-1R expression in the GI tract is classically ascribed to the myenteric plexus, the highest level of GLP-1R expression in the gut is in the Brunner's glands in the upper duodenum (73, 87); it is, in fact, one of the highest GLP-1R-expressing organs in humans. While little is known about the role of this GLP-1R population, a function of the Brunner's glands is to secrete mucus to lubricate the intestinal wall and, as GLP-1 has been suggested to be a positive regulator of gut-barrier function, GLP-1 may act on these glands to increase mucus secretion, thereby decreasing inflammation and protecting against intestinal damage. Another GLP-1R population that may have an important role in the GI tract is found on intestinal intraepithelial lymphocytes; these receptors have also been implicated in positively regulating gut-barrier function and inflammation (98). Parietal cells also show fairly strong expression of the GLP-1R (73), and GLP-1 has been shown to inhibit gastric acid secretion in humans, although the importance of this effect is currently unclear (99).

### Heart

In the human and non-human primate heart, the GLP-1R is localized to myocytes of the sinoatrial (SA) node (73). This receptor population may account for the rapid and sustained, but relatively small, increase in pulse rate with GLP-1RAs in humans (100, 101). The physiological relevance of this increase in pulse rate is yet to be elucidated. The heart is another organ where species-specific differences in GLP-1R expression may be evident. While initial studies described GLP-1R expression in the cardiomyocytes of rodents (102), it was later shown that, in general, these cells do not express the GLP-1R (103). In rodents, the GLP-1R is now known to be expressed in the atrium but, unlike in humans and non-human primates, not in in the SA node (73, 103). While the resulting effect may be the same, the exact molecular mechanism underlying the GLP-1-induced regulation of heart rate (and blood pressure, BP) in rodents and humans most likely differs. Also, humans may have a variable and disease-specific GLP-1R expression in the heart (104).

### Lung and Kidney

**Figure 6:**
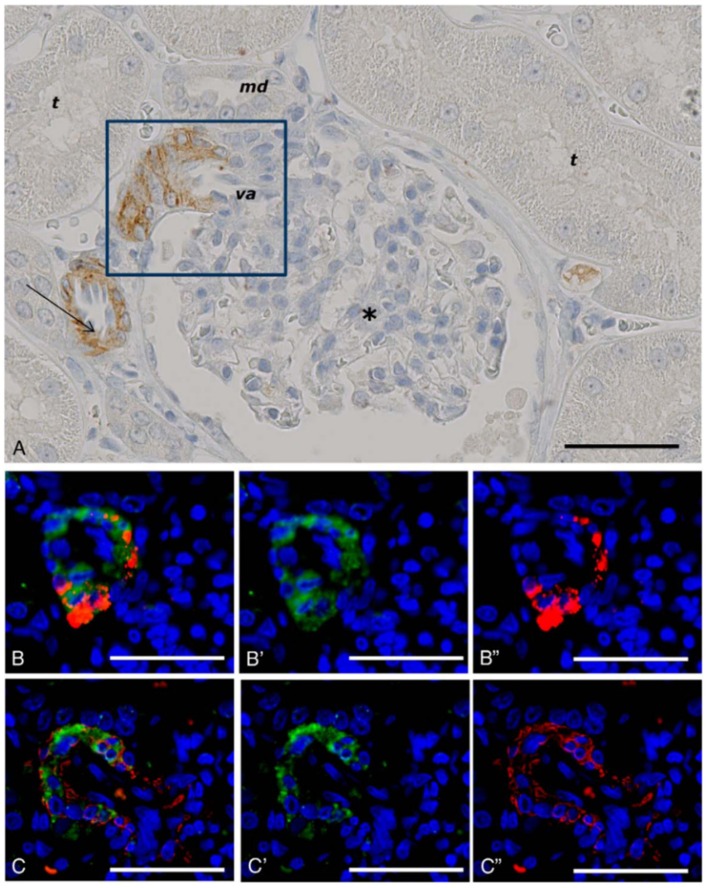
GLP-1 receptor immunoreactivity in non-human primate smooth muscle cells. **(A)** GLP-1R immunoreactivity in non-human primate smooth muscle cells of a vas afferens (va) arteriole at the vascular pole. Note the absence of signal in the macula densa (md), tubuli (t), glomerulus (^*^), and endothelial cells within an arteriole (arrow). **(B,C)** Near-adjacent sections showing double labeling for GLP-1R (green)/renin (red) and GLP-1R (green)/SMA (red), respectively. **(B′,C′)** Same as **(B,C)**, respectively, with red fluorescence digitally removed to highlight GLP-1R immunoreactivity. **(B″,C″)** Same as **(B,C)**, respectively, with green fluorescence digitally removed to highlight renin and SMA immunoreactivity, respectively. Scale bars correspond to 50 μm.GLP-1R, glucagon-like peptide 1 receptor; SMA, smooth muscle actin. Reproduced with permission from Pyke et al. (73). By permission of Oxford University Press on behalf of the Endocrine Society, available at: https://academic.oup.com/endo/article/155/4/1280/2423090?searchresult=1. This figure is not included under the CC-BY license of this publication. For permissions, please contact journals.permissions@oup.com.

Our findings using non-human primate kidney tissue, as shown in Figure 6, demonstrate that GLP-1Rs are exclusively expressed in smooth muscle cells in the walls of arteries and arterioles, and studies with human tissue show the same (73, 87). These cells are also positive for renin (73). In the lung, it also seems that only smooth muscle cells express the GLP-1R (73, 87). The roles of these receptor populations are, however, unclear. GLP-1 has been proposed to have a role in lung protection (105), although this has primarily been described in rodents, where the GLP-1R is expressed at higher levels (87) and also in more diverse cell types (e.g., type 2 pneumocytes) than in humans (106). In addition to a potential role in local organ protection (107, 108), the kidney smooth muscle cell GLP-1Rs may have a role in regulating systemic BP via the renin–angiotensin pathway (103).

### Liver

Hepatocytes do not express any measurable level of GLP-1Rs (73, 74). Unfortunately, there are numerous reports of such expression, based on findings with non-specific antibodies in IHC studies (76). Despite the lack of GLP-1R expression, GLP-1RAs appear to have a positive effect on non-alcoholic fatty liver disease/non-alcoholic steatohepatitis (NAFLD/NASH), as evidenced by a clinical trial with liraglutide and now under further investigation in clinical trials with semaglutide (109, 110). Our recent animal study with semaglutide confirmed the lack of GLP-1R expression in the liver, and indicated the mechanism for improving NAFLD/NASH to be indirect, via reduced inflammation (111).

### Thyroid

As noted above, the thyroid gland is an organ where species-specific expression of the GLP-1R is evident. In rodents there is high expression of GLP-1Rs in the calcitonin-producing C-cells of the thyroid, and activation of these receptors by GLP-1RAs stimulates the adenylate cyclase pathway, leading to upregulation of calcitonin synthesis and, subsequently, hyperplasia and an increased incidence of adenomas (88). In contrast to rodents, non-human primates have fewer C-cells and no measurable activation by GLP-1RAs (88). Similarly, humans have little, if any, GLP-1R expression in the thyroid (85). The findings in rodents appear, therefore, to be of much lesser relevance in humans (112, 113).

### Brain

Brain-derived GLP-1 is primarily produced in specific populations of neurons expressing the GLP-1 precursor, preproglucagon (PPG) in the nucleus tractus solitarus (NTS) of the hindbrain and project to multiple brain regions that express the GLP-1R. These findings from our studies are summarized in Table 2, which outlines our detailed analysis of GLP-1R expression in the rodent brain (115). GLP-1 is likely best described as a neuropeptide with physiologically and pharmacologically relevant effects on food intake and body weight (116, 117), potential neuromodulatory roles, and possible effects in a range of other neuropathological conditions including neurodegenerative diseases (e.g., Alzheimer's, Parkinson's), brain trauma and stroke, as well as in depression, anxiety and addiction (118–123). GLP-1Rs are expressed in a variety of discrete areas of the brain. The GLP-1R is most abundantly expressed in the hypothalamus, the brain stem and the septal nucleus, where mRNA expression is also seen. Many other neurons express the GLP-1R, but less abundantly, and neuronal projections also express receptors. We previously demonstrated a similar expression pattern between rodents and non-human primates (86). Although there appears to be a high degree of overlap in GLP-1R expression in the rodent and non-human primate brain, one notable difference may be an apparent higher expression of GLP-1R in the central amygdala and bed nucleus of the stria terminalis of non-human primates (86). While caution should be exercised, as this comparison is based on separate studies, this finding, if correct, may indicate a more important effect of GLP-1 in these areas in primates, including humans (86).

**Table 2:** Summary of GLP-1 receptor location in the mouse brain and comparison with external references.

**Structure**		**Area**	**Protein**	**mRNA**	**Cork et al. (114) (Protein)**	**AIBS mRNA**
Telencephalon	ACB	Nucleus accumbens	+	+	+	+
	AOB	Accessory olfactory bulb	+	+	+	+
	BMA	Basomedial amygdalar nucleus	+	–	+	+
	BST	Bed nucleus of stria terminalis	+	+	+	+
	CeA	Central amygdala	+	+	+	+
	CeA/AAA	Central amygdala/anterior amygdalar area	+	+	+	+
	COA	Cortical amygdalar area	+	–	+	+
	DG	Dentate gyrus	+	+	+	+
	LPO	Lateral preoptic area	+	+	+	+
	LS	Lateral septal nucleus	+	+	+	+
	NLOT/COA	Nucleus of the lateral olfactory tract/cortical amygdalar area	+	–	+	+
	OV	Vascular organ of the laminae terminalis	+	NA	+	NA
	PA	Posterior amygdalar nucleus	+	–	+	+
	SF	Septofimbrial nucleus	+	+	NA	+
	SFO	Subfornical organ	+	+	+	+
	TRS	Triangular nucleus of septum	–	+	NA	+
	TT	Tenia tecta	+	+	+	+
Diencephalon	ARH	Arcuate hypothalamic nucleus	+	+	+	+
	DMH	Dorsomedial hypothalamic nucleus	+	+	+	+
	GENd	Geniculate group, dorsal thalamus	+	–	+	+
	Hb	Habenula	+	–	+	+
	LHA	Lateral hypothalamic nucleus	+	+	+	+
	ME	Median eminence	+	–	NA	+
	PH	Posterior hypothalamic nucleus	+	–	+	+
	PP	Peripeduncular nucleus	+	+	NA	+
	PVH	Paraventricular hypothalamic nucleus	+	+	+	+
	RCH	Retrochiasmatic area	+	–	+	+
	SO	Supraoptic nucleus	+	+	NA	+
	TU	Tuberal nucleus	+	+	NA	+
	ZI	Zona incerta	+	+	+	+
Mesencephalon	APN	Anterior pretectal nucleus	+	+	+	+
	DR	Dorsal raphe	+	–	NA	+
	MM	Medial mammillary nucleus	+	–	+	+
	MPT	Medial pretectal area	+	+	+	+
	MRN	Midbrain reticular nucleus	+	–	NA	+
	PAG	Periaqueductal gray	+	+	+	+
	PPT	Posterior pretectal area	+	+	+	+
	SCs	Superior colliculus, sensory related	–	+	NA	+
	SCm	Superior colliculus, motor related	+	+	NA	+
	SCTV	ventral spinocerebellar tract	+	–	NA	+
	SNr	Substantia nigra, reticular part	+	+	NA	+
	SNc	Substantia nigra, compact part	+	+	NA	+
Pons	NI	Nucleus incertus	+	–	NA	+
	NLL	Nucleus of the lateral lemniscus	+	–	NA	+
	PB	Parabrachial nucleus	+	+	NA	+
	PCG	Pontine central gray	+	–	NA	+
	PG	Pontine gray	+	+	NA	+
	PRNc	Pontine reticular nucleus, caudal part	–	+	NA	+
	PRNr	Pontine reticular nucleus	–	+	NA	+
	PSV	Principal sensory nucleus of the trigeminal	+	+	NA	+
	SOC	Superior olivary complex	+	–	NA	+
Medulla	AMB	Nucleus ambiguus	–	+	NA	+
	AP	Area postrema	+	+	+	+
	CU	Cuneate nucleus	+	+	NA	+
	DMX	Dorsal motor nucleus of the vagus nerve	+	–	NA	+
	ECU	External cuneate nucleus	+	+	NA	+
	FL	Flocculus (cerebellum)	+	–	NA	+
	IO	Inferior olivary complex	+	+	+	+
	LRNm	Lateral reticular nucleus, magnocellular part	+	+	NA	+
	MARN	Magnocellular reticular nucleus	–	+	NA	+
	MDRN	Medullary reticular nucleus	–	+	NA	+
	MV	Medial vestibular nucleus	+	+	NA	+
	NTS	Nucleus tractus solitarus	+	+	+	+
	PARN	Parvocellular reticular nucleus	–	+	NA	+
	SPVC	Spinal nucleus of the trigeminal, caudal part	+	+	NA	+

*Presence (+) or absence (–) of GLP-1R protein and mRNA in mouse brain. The two right-hand columns indicate whether GLP-1R was observed as a protein or mRNA (AIBS). AIBS, Allen Brain Atlas; GLP-1R, glucagon-like peptide 1 receptor; NA, not assessed/reported. Source: Jensen et al. (115)*.

## The Pharmacology of Liraglutide and Semaglutide

Liraglutide and semaglutide are both long-acting GLP-1RAs that, despite differing administration intervals and doses, have pharmacodynamic (PD) effects for 24 h/day (124, 125). Liraglutide was developed for OD administration, and is available as a OD injection of up to 1.8 mg (126), whereas semaglutide is available as a OW injection of up to 1.0 mg (127)—both for the treatment of T2D. Liraglutide is additionally approved for the treatment of obesity, at a dose of 3.0 mg (128), while semaglutide is in clinical development as a treatment for obesity (phase 3) and NASH (phase 2). Furthermore, phase 3 clinical trials in T2D are ongoing with semaglutide administered orally (in a co-formulation with an absorption enhancer); mechanistic findings with this formulation are summarized toward the end of this article. The most common side effect of the GLP-1RA class is GI-related adverse events (AE) including nausea, diarrhea, and vomiting (129) which are dose-dependent and typically present in the up-titration phase. Most GI AEs with GLP-1RAs are mild and transient in nature but, in some patients, they can lead to premature treatment discontinuation. Hence, both liraglutide and semaglutide dosing should be titrated initially (126, 127).

Using fatty acids engineered onto a peptide, thereby facilitating binding to albumin, is a novel concept for drug protraction. Eleven fatty-acid binding sites are described on human albumin (11–13) and, as the human albumin concentration is ~0.6 mM (5) and the exposure levels of liraglutide and semaglutide are in the 20–40 nM range (125, 130), there appears to be a large surplus in binding capacity on albumin. Consequently, no apparent safety issues have arisen from this approach. No interactions with other albumin-binding drugs have been shown (131, 132), likely because most other drugs do not bind to the fatty-acid binding sites. As patients with liver deficiencies can have lower levels of plasma albumin, liraglutide, and semaglutide were evaluated in this population; studies showed no differences in PK and, as such, no dose adjustment is required (133, 134). Likewise, studies have shown that no dose adjustment of liraglutide or semaglutide is needed in patients with renal deficiencies (135, 136); neither drug is cleared via the kidney (44, 125). Liraglutide, which comprises natural amino acids and a C16 mono-acid, is fully metabolized in the same way as other peptides and fatty acids (44). Semaglutide, which contains an amino acid described in nature but not in humans (Aib) and a more synthetic component in the spacer region, is still fully metabolized (125). Indirect comparisons indicate the only difference to be that more semaglutide metabolites are excreted in feces compared with liraglutide (125).

Clinical efficacy studies evaluated liraglutide and semaglutide with regard to glucose lowering, weight loss and CV risk reduction. These effects are described below, with evidence from clinical trials and animal studies presented; the latter where it adds to our understanding.

### Glucose Lowering

Liraglutide was the first long-acting GLP-1RA to become available for the treatment of T2D, receiving market authorization in 2009 in the EU (137), 4 years after the short-acting GLP-1RA, exenatide, was first approved in the US (138). Semaglutide was approved in 2017 (139). The pharmacology of short- and long-acting GLP-1RAs differ (140). Short-acting GLP-1RAs are administered before a meal, and have a greater effect on gastric emptying and postprandial glucose, primarily after the meal, whereas long-acting GLP-1RAs have less of an effect on gastric emptying and postprandial glucose excursions, but a more pronounced effect on fasting blood glucose and weight loss (140). These differences were first illustrated in LEAD and AWARD, the phase 3 clinical trial programs for liraglutide and dulaglutide, respectively (141, 142).

While semaglutide was primarily designed as a longer acting compound compared with liraglutide (53), it has been shown to provide benefits beyond its protraction; studies have demonstrated greater glucose-lowering and weight-loss effects with semaglutide compared with other GLP-1RAs, including liraglutide (101, 143, 144).

The glucose-lowering mechanisms of action of GLP-1RAs are well-characterized. Initially GLP-1 was described as an incretin hormone that stimulates glucose-dependent insulin secretion, increases insulin biosynthesis, improves beta-cell function and acts as a glucagonostatic regulator (145–148). Physiologically, however, the primary effect of GLP-1 is most likely the reduction of gastric emptying, with the effects on insulin and glucagon being of lesser relevance (95).

There has been some controversy regarding the glucagonostatic effect of GLP-1RAs, and whether such an effect exists. Some studies have indicated a neutral, or even an increased, response. The challenge may lie in measuring glucagon, which can be very difficult (149–151). For semaglutide, however, the reductions in glucagon (both postprandial and fasting) are clear. Indeed, plasma glucagon was shown to be significantly reduced at week 56 with semaglutide vs. sitagliptin or exenatide ER (143, 152).

Increased beta-cell function and insulin biosynthesis has been demonstrated for both liraglutide and semaglutide, with favorable proinsulin/insulin ratios compared with other glucose-lowering agents (124, 141, 143, 152–155); this includes sulfonylureas, which have been shown to increase insulin secretion with no effect on insulin biosynthesis (156). In the late 1980s/early 1990s, several studies demonstrated that GLP-1 increased beta-cell mass, prevented beta-cell death and, potentially, induced neogenesis (157–160). While the effects on beta-cell mass, apoptosis and proliferation have also been demonstrated with liraglutide in young rodents (161, 162), they have not been shown to translate into a clinically significant effect in terms of the permanent prevention of diabetes progression, most likely because the effects on beta-cell mass are specific to younger animals and human beta-cells have a much lower, if any, capacity for regeneration (163, 164). The effect on insulin biosynthesis, initially described in 1987 (147), does seem to translate into clinical data, as measured by a reduction in the proinsulin/insulin ratio, as mentioned above.

Insulin sensitivity appears not to be directly affected by GLP-1RAs. Clinical studies with semaglutide indicate an effect on increased insulin sensitivity, likely mediated by semaglutide-induced weight loss. Reductions in insulin resistance were shown to be greater with semaglutide (−27 to −46%) vs. placebo (−17%), sitagliptin (−28%), or exenatide ER (−28%), and a *post-hoc* analysis indicated that reductions with semaglutide were primarily mediated by weight loss (143, 152, 165–167).

### Weight Loss

Several studies document the weight-loss effect and mechanism of liraglutide and semaglutide: these are outlined below. Clinical data have shown liraglutide to provide greater reductions in body weight compared with short- and long-acting exenatide, short-acting lixisenatide and long-acting dulaglutide, with markedly greater reductions vs. albiglutide (141, 155, 168–170). Trials with liraglutide 1.8 mg have shown weight loss up to 3.6% (3.3 kg) at 26 weeks and 4.7% (5.0 kg) at 56 weeks in subjects with T2D (171–173), while trials with liraglutide 3.0 mg have shown weight loss of between 7.9% (8.9 kg) and 8.2% (7.3 kg) at 56 weeks in subjects with obesity (173–175). Furthermore, with a run-in phase of a low-calorie diet, total weight loss of up to 12.5 kg can be achieved (of which half [6.2%] was observed during the liraglutide 3.0 mg treatment period) in subjects with obesity (176). This difference in weight loss between subjects with T2D vs. subjects with obesity may be due to the indirect effect of improved glucose control on body weight homeostasis. Semaglutide has, subsequently, been shown to provide greater weight loss than liraglutide and other GLP-1RAs, with a comparable safety profile (101, 143, 177). While it could be argued that these findings may be different if all GLP-1RAs were dose optimized in the same way, this seems unlikely given that semaglutide-induced weight loss is about two-fold greater than liraglutide (177) and three-fold greater than exenatide (143).

It has been known since the late 1990s that GLP-1 lowers food intake; studies in rodents published in 1996 demonstrated that intracerebroventricular injection of GLP-1 had a marked effect in both fasting and fed states, and a publication in 1995 showed that a rodent tumor model that produced glucagon and GLP-1 was associated with severe anorexia (116, 178, 179). Subsequent clinical studies with liraglutide and semaglutide confirmed these findings, and further demonstrated the effect to be mediated via reduced energy intake. Liraglutide showed a 16% reduction in energy intake vs. placebo over 5 weeks, with no effect on energy expenditure (180). Subjects with obesity treated with semaglutide for 12 weeks showed an average 24% reduction in energy intake over 3 meals/snacks and, similar to liraglutide, no effect on energy expenditure despite a decrease in resting metabolic rate (RMR). As RMR did not increase with semaglutide, it may be inferred that weight loss was likely as a result of reduced energy intake (181). Also, in a study with liraglutide, where 24-h respiratory chambers were used, liraglutide increased fat oxidation (180). Furthermore, semaglutide had a positive effect on the hedonic aspects of food intake such as reduced cravings, increased ability to control food intake and an altered food preference (i.e., a lower liking for high-fat and non-sweet foods) (181). The lower preference for high-fat foods, which are typically more energy dense, may explain the reduction in calorie intake with semaglutide. Together these effects indicate an impact on both energy homeostasis and the hedonic aspects and reward-related behaviors pertaining to food.

A number of hypotheses have been explored to explain the effect of GLP-1RAs on energy intake including: GI AEs, gastric emptying and effects (via the gut and directly) on the central nervous system.

While GI side effects have been suggested to be involved in mediating the weight-loss effect of GLP-1RAs, analysis of large clinical trial data sets with liraglutide and semaglutide has shown that, while GI side effects may lead to more initial weight loss, they are not the mechanism of weight loss as many patients have weight loss and no side effects (182, 183).

It has also been proposed that GLP-1RAs affect appetite and energy intake via gut-to-brain communication (184). While there is no doubt that the vagus nerve and dorsal motor complex express GLP-1Rs, it is less clear if this is the receptor population via which GLP-1RAs mediate reduced energy intake (185–187). A study assessing the acute effect of liraglutide in vagotomized subjects did implicate the vagus nerve in mediating the appetite effect (188), but the sample size in the study was small and subjects had a very low appetite at baseline, making it difficult to draw conclusions. Gastric emptying has also been proposed as a mechanism by which GLP-1RAs affect energy intake, similar to the suggestion for glucose-lowering. However, since long-acting GLP-1RAs like liraglutide and semaglutide have little effect on gastric emptying, this seems unlikely to be the mechanism by which all GLP-1RAs reduce energy intake and mediate weight loss (140). Although no clinical studies have directly compared gastric emptying with long- and short-acting GLP-1RAs, their relative effects were illustrated in an animal study comparing liraglutide and exenatide, where the latter was shown to have a profound effect on gastric emptying while liraglutide had very little effect (124, 189). These differences in gastric emptying do appear to translate into the clinical setting (35, 180, 190–192), as exemplified by prandial glucose data. However, the relationship is the opposite for weight loss, with more weight loss observed with long-acting GLP-1RAs despite the lesser effect of these drugs on gastric emptying. It may be that short-acting GLP-1RAs act more like endogenous GLP-1 and communicate to the brain primarily via the vagus nerve, whereas long-acting GLP-1RAs like liraglutide and semaglutide primarily access the brain directly.

## Effects in the Brain, Weight Loss and Beyond?

**Figure 7:**
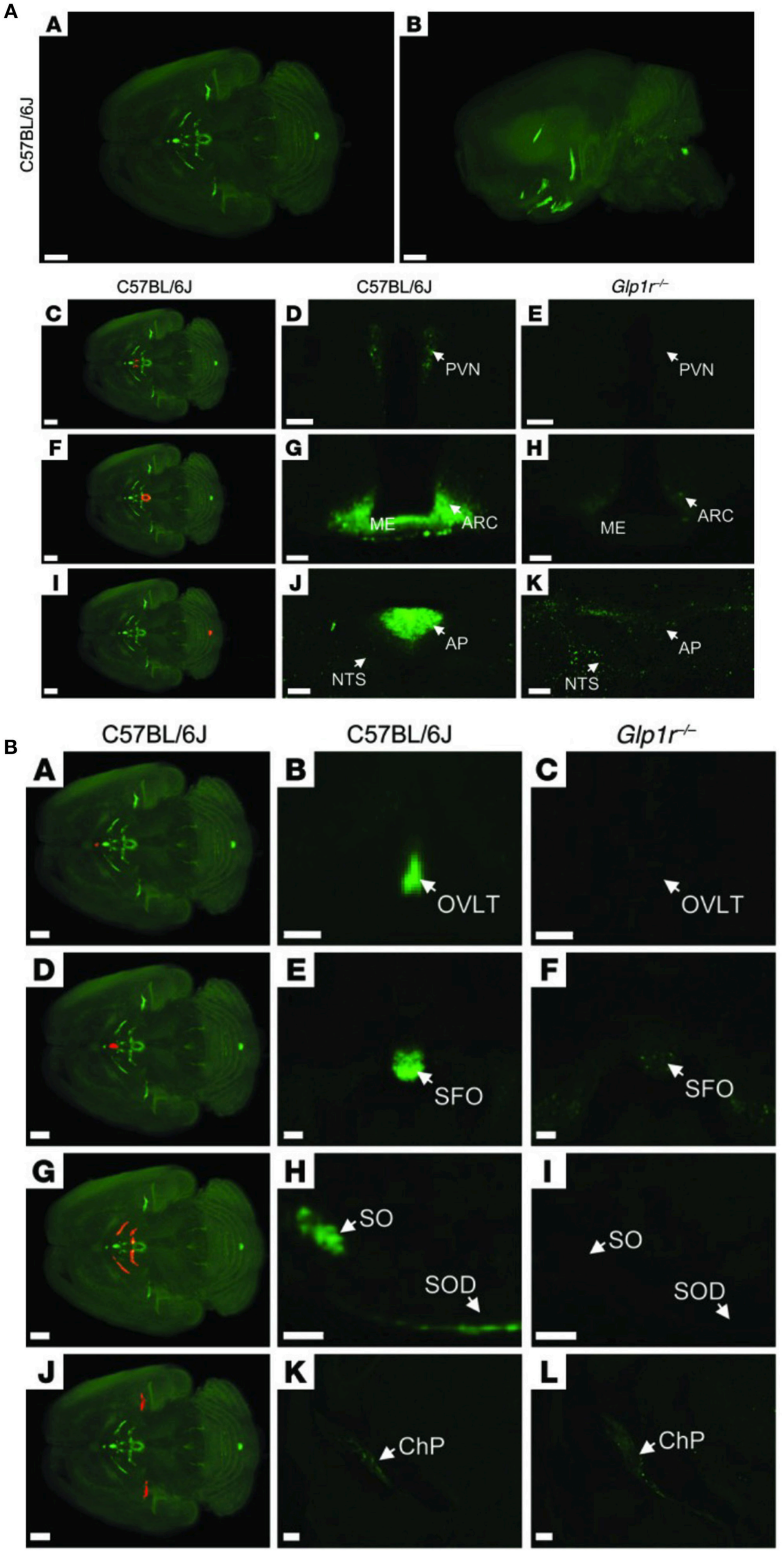
Distribution of fluorescently labeled liraglutide in the mouse brain. **(A)** Representative whole brain images viewed in the (A) dorsoventral or (B) sagittal plane from C57BL/6J mice administered with liraglutide^750^. The brain tissue was scanned at 620 and 710 nm, representing both autofluorescence from the tissue (gray) and specific signal (green). The red regions in (C,F,I) are shown at higher magnification in (D,G,J), respectively. Images in (D,E,G,H,J,K) show high-magnification views of a single section from (D,G,J) C57BL/6J or (E,H,K) Glp1r^−/−^mice administered liraglutide^750^. Liraglutide^750^ was detectable in (C,D) PVN, (F,G) ME and ARC, and (I,J) AP. (E,H,K) In mice lacking a functional GLP-1R, no liraglutide^750^ signal could be detected in any of these regions. Scale bars: 200 μm (A,B,C,F,I); 50 μm (D,E); 100 μm (G,H,J,K). **(B)** The brain tissue was scanned at 620 and 710 nm, representing both autofluorescence from the tissue (gray) and specific signal (green). The red regions in (A,D,G,J) are shown at higher magnification in (B,E,H,K), respectively (unspecific staining has been removed). Images in the middle and right columns represent enlargements of a single section from (B,E,H,K) C57BL/6J or (C,F,I,L) Glp1r^−/−^ mice administered with liraglutide^750^. Liraglutide^750^ was detectable in (A,B) organum vasculosum of the lamina terminalis (D,E), subfornical organ (G,H), supraoptic nucleus and supraoptic decussation, and (J,K) ChP. (C,F,I,L). In mice lacking a functional GLP-1R, no liraglutide^750^ signal could be detected in any of these regions except from ChP. Scale bars: 200 μm (A,D,G,J–L); 100 μm (B,C,E,F,H,I). AP, area postrema; ARC, arcuate nucelus; ChP, choroid plexus; ME, median eminence; NTS, nucleus of the solitary tract; OVLT, organum vasculosum of lamina terminalis; PVN, paraventricular nucleus; SFO, subfornical organ; SO, supraoptic nucleus; SOD, supraoptic decussation. Republished with permission of American Society For Clinical Investigation, from Secher et al. (117), Copyright 2018; permission conveyed through Copyright Clearance Center, Inc.

**Figure 8:**
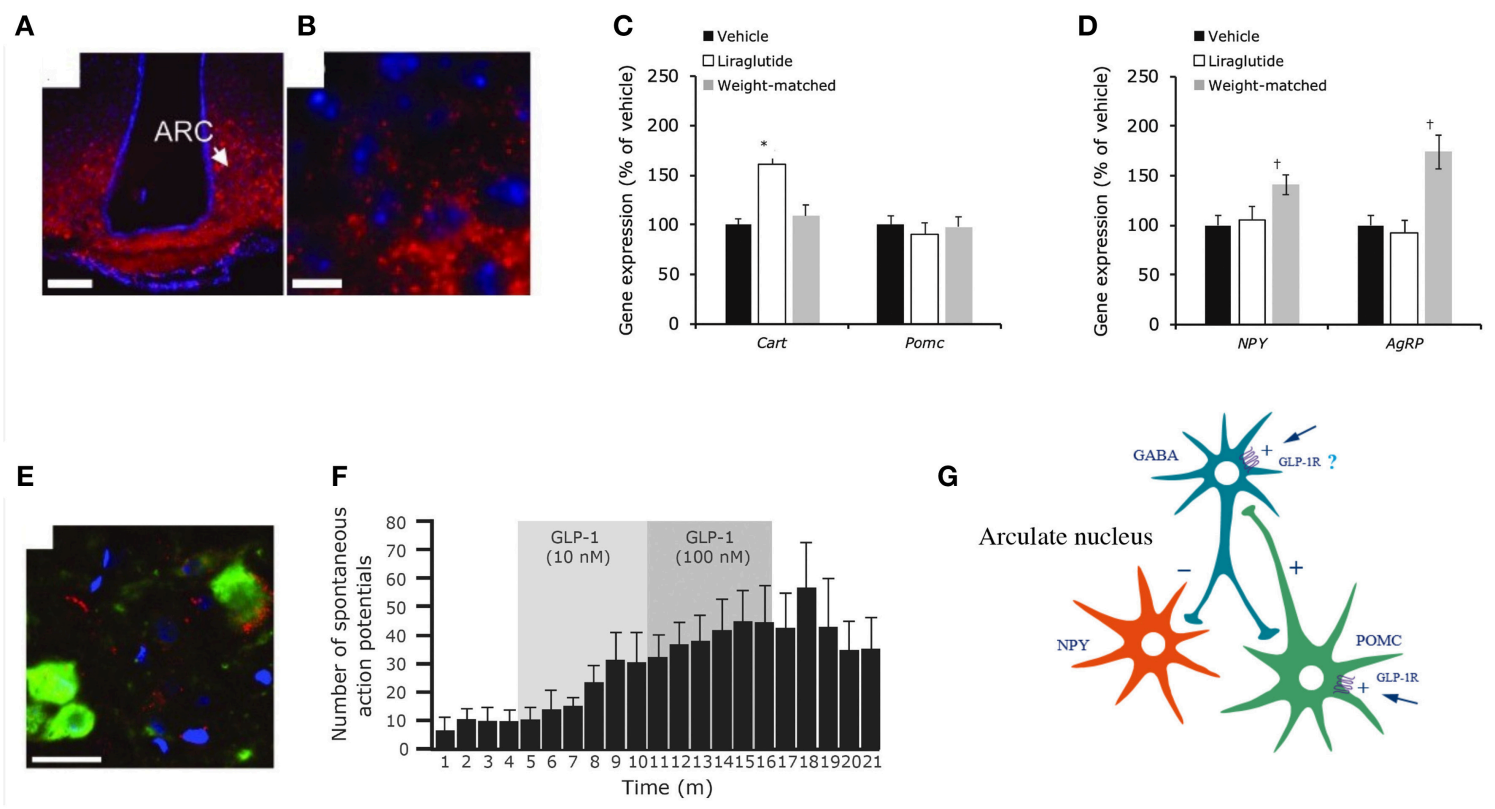
Liraglutide and the mouse brain. Distribution of liraglutide^594^ or exendin(9-39)^594^ in brain. **(A,B)** liraglutide^594^ had access to ARC, in which it bound the GLP-1R and internalized. **(B)** High-magnification image showed that liraglutide^594^ was internalized and the fluorescent signal was located in the cytoplasm. Staining, Hoechst nuclear stain (blue) and liraglutide^594^/exendin(9-39)^594^(red). Scale bars: 100 μm. Liraglutide treatment regulates ARC gene expression and ARC neuronal activity. **(C)** Liraglutide treatment for 28 days in DIO rats significantly increased mean *Cart* mRNA levels in the ARC (^*^p < 0.001 liraglutide vs. vehicle and vs. weight matched), whereas *Pomc* expression was unaffected. **(D)** *Npy* and *Agrp* mRNA levels were significantly increased in weight-matched rats–but not following treatment with liraglutide (†*p* < 0.05 weight matched vs. vehicle and vs. liraglutide). Data are mean ± SEM, and statistical analyses were performed using 1-way ANOVA, with Fisher's *post hoc* test. Images **(E,F)** show neuronal accumulation and activity following GLP-1R simulation. Specifically, panel **(E)** shows CART- and liraglutide^594^-positive cells shown as mean ± SEM. Staining, Hoechst nuclear strain (blue), liraglutide594 (red) and CART (green). Scale bar: 25 μm. Panel **(F)** shows the effects of GLP-1(7-36)amide on firing rate of spontaneous action potentials in POMC/CART neurons. **(G)** Proposed regulation of neuronal activation by liraglutide. Summary diagram demonstrating the suggested regulatory pathway of GLP-1 on ARC NPY and POMC neurons. GLP-1 stimulates POMC neurons directly through the GLP-1R and is suggested to indirectly inhibit ARC-NPY neurons through a local inhibitory GABA neuron. ANOVA, analysis of variance; ARC, arcuate nucleus; CART, cocaine- and amphetamine-regulated transcript; DIO, diabetes induced obesity; GABA, gamma-amino butyric acid; GLP-1, glucagon-like peptide-1; GLP-1R, glucagon-like peptide-1 receptor; NYP, neuropeptide Y; POMC, proopiomelanocortin; SEM, standard error of the mean; veh, vehicle. Republished with permission of American Society For Clinical Investigation, from Secher et al. (117), Copyright 2018; permission conveyed through Copyright Clearance Center, Inc.

Animal studies show that GLP-1Rs located in the brain are responsible for mediating the effect of liraglutide on weight loss (185). In one animal study, knock-down of GLP-1Rs in the brain neurons almost eliminated the effect of liraglutide, whereas knock-down of GLP-1Rs in the peripheral nervous system did not alter its efficacy significantly (185). Our studies showed that the effect of liraglutide on body weight was not compromised in vagotomized animals, supporting the concept that GLP-1Rs in the brain, and not gut-to-brain communication, is mostly responsible for the weight loss effect (117). Pinpointing precisely which GLP-1R populations in the brain are necessary for appetite regulation is, however, challenging. Our studies show that liraglutide enters the brain in a GLP-1R-dependent manner (117). As shown in Figure 7, a fluorescent version of liraglutide was injected subcutaneously into mice and tracked using single plane illumination microscopy (SPIM). In addition to a number of circumventricular organs [CVOs, e.g., median eminence (ME), area postrema (AP), subfornical organ, organum vasculosum of lamina terminalis], liraglutide was also observed in the hypothalamus [arcuate nucleus (ARC), paraventricular nucleus (PVN), supraoptic NTS, supraoptic decussation], which would have necessitated its uptake across the blood–brain barrier. It is hypothesized that uptake could be via fenestrated capillaries and through ME transport processes, specifically tanycytes, similar to findings with leptin (193–195). So, peripherally dosed long-acting GLP-1RAs have the ability to access neurons in both the CVOs and in a few select sites in the hypothalamus, but do not broadly cross the blood brain–barrier. In the hypothalamus, liraglutide directly accessed the proopiomelanocortin/cocaine- and amphetamine-regulated transcript (POMC/CART) neurons expressing the GLP-1R, and appeared to be internalized, as expected when a GPCR ligand activates its receptor (117). Additionally, there was strong liraglutide uptake in the ME. *In vivo* dosing of liraglutide in animals, followed by gene expression analysis of the hypothalamus, documented an increase in CART, but not POMC, mRNA and the inhibition of increases in neuropeptide Y/agouti-related peptide. Further *ex vivo* staining showed overlap of liraglutide uptake with CART immunoreactivity and activation of the neurons, as measured by an increase in membrane potential and the number of spontaneous action potentials (117). The effects on the hypothalamic appetite signals, as depicted in Figure 8, offer a hypothesis on how liraglutide increases satiety and reduces feelings of hunger in patients. The effects in the hypothalamus are unlikely to be the only way in which liraglutide affects signaling related to feeding. This is supported by a study showing that re-introduction of GLP-1Rs into the POMC neurons of GLP-1R knockout mice did not reinstate weight loss in lean animals, but partially restored weight loss (~50%) in obese animals (196). Another study demonstrated that the overall effect of liraglutide requires GLUT neurons, but not GABAergic neurons (197). Given the complexity of neuronal networks and that numerous areas of the brain involved in feeding express GLP-1Rs (198), it is plausible that many of the GLP-1R populations in the brain are involved, but may not be entirely essential, in mediating the effect of GLP-1RAs on energy intake. This suggests that GLP-1R-mediated food-intake control pathways have a degree of redundancy, supported by the fact that GLP-1R null animals exhibit limited phenotypic consequences (199). Studies in genetically engineered animals may thus underestimate the number of GLP-1R populations in the brain involved in feeding, because, for illustration, it is intrinsically difficult to show whether a sub-population of brain GLP-1Rs mediates 10 or 20% of an effect.

**Figure 9:**
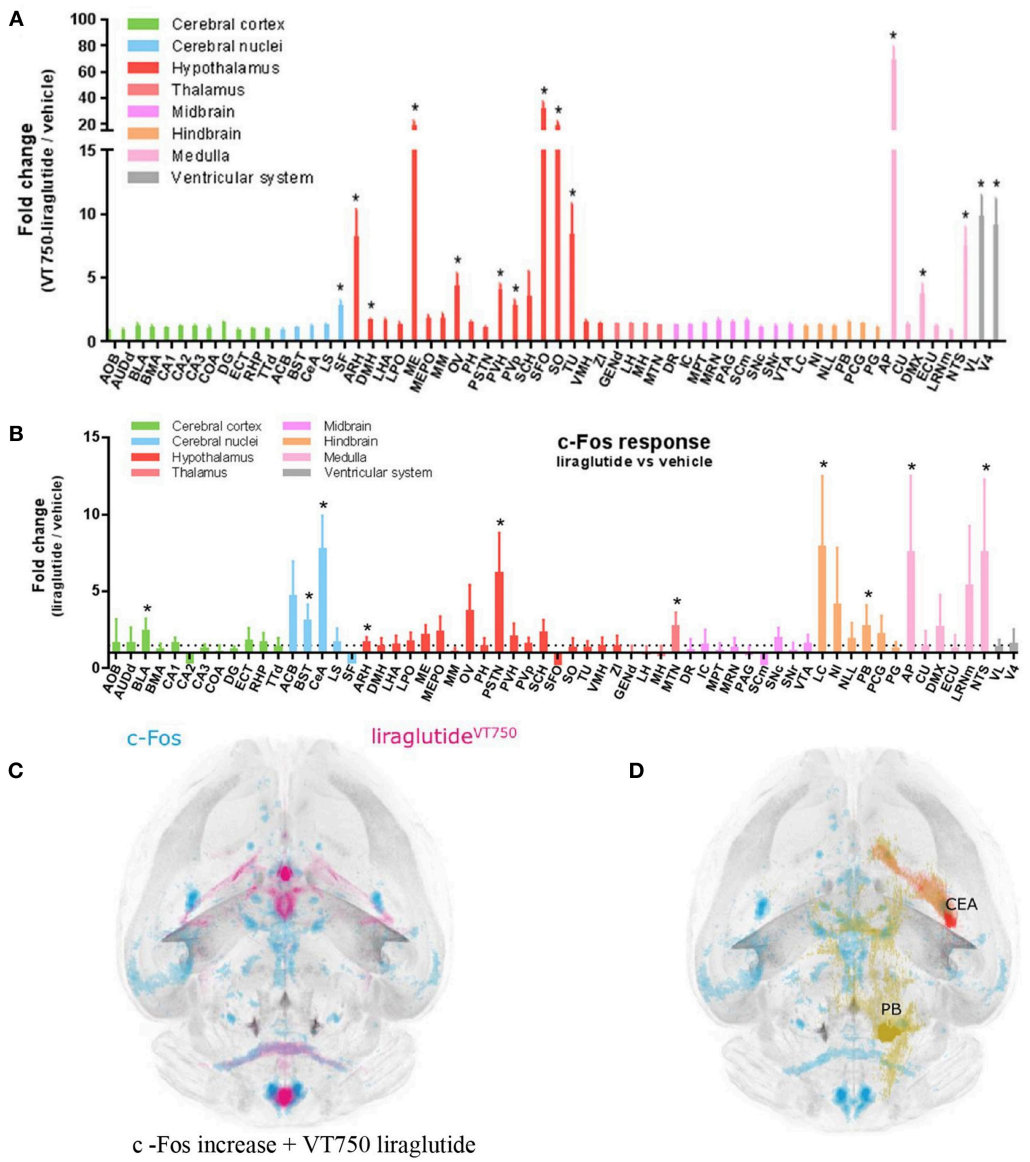
Liraglutide, cFOS, and the hindbrain. Brain region with liraglutide^VT750^ access. Bar graph **(A)** shows the mean fold change and standard deviation (SD) of the total fluorescence signal in selected brain regions comparing liraglutide^VT750^ and vehicle (*n* = 5). An asterisk marks significant difference between treatments when analyzed in individual brain regions using a false discovery rate value of 5% to correct for multiple comparisons. Note the split y-axis when interpreting result and standard deviation. Neural activation following liraglutide administration. Bar graph **(B)** shows the mean fold change SD to total c-Fos heat map signal in selected brain regions comparing liraglutide- and vehicle-dosed animals. Regions were selected as having either liraglutide^VT750^ access, GLP-1R expression, or c-Fos response. An asterisk marks significant difference between treatments when analyzed in individual brain regions using a false discovery rate value of 20% to correct for multiple comparisons. **(C)** Liraglutide-specific c-Fos increase overlaid with the average liraglutide^VT750^ distribution from bar graph **(B)**. **(D)** Connectivity maps visualized by horizontal maximum intensity projection overlaid with the average c-Fos increase following liraglutide administration from **(C)**. ACB, nucleus accumbens; AOB, accessory olfactory bulb; AP, area postrema; ARH, arcuate hypothalamic nucleus; AUDd, dorsal auditory area; BLA, basolateral amygdalar nucleus; BMA, basomedial amygdalar nucleus; BST, bed nuclei of the stria terminalis; CA (1, 2, and 3), field CA(1, 2, and 3); CeA, central amygdalar nucleus; COA, cortical amygdalar area; CU, cuneate nucleus; DG, dentate gyrus; DMH, dorsomedial nucleus of the hypothalamus; DMX, dorsal motor nucleus of the vagus nerve; DR, dorsal nucleus raphe; ECT, ectorhinal area; ECU, external cuneate nucleus; GENd, geniculate group, dorsal thalamus; IC, inferior colliculus; LC, locus ceruleus; LH, lateral habenula; LHA, lateral hypothalamic area; LPO, lateral preoptic area; LRNm, lateral reticular nucleus, magnocellular part; LS, lateral septal nucleus; ME, median eminence; MEPO, median preoptic nucleus; MH, medial habenula; MM, medial mammillary nucleus; MPT, medial pretectal area; MRN, midbrain reticular nucleus; MTN, midline group of the dorsal thalamus; NI, nucleus incertus; NLL, nucleus of the lateral lemniscus; NTS, nucleus of the solitary tract; OV, vascular organ of the lamina terminalis; PAG, periaqueductal gray; PB, parabrachial nucleus; PCG, pontine central gray; PG, pontine gray; PH, posterior hypothalamic nucleus; PSTN, parasubthalamic nucleus; PVH, paraventricular hypothalamic nucleus; PVp, periventricular hypothalamic nucleus, posterior part; RHP, retrohippocampal region; SCm, superior colliculus, motor related; SCH, suprachiasmatic nucleus; SF, septofimbrial nucleus; SFO, subfornical organ; SNc, substantia nigra, compact part; SNr, substantia nigra, reticular part; SO, supraoptic nucleus; TTd, taenia tecta, dorsal part; TU, tuberal nucleus; VL, lateral ventricle; VMH, ventromedial hypothalamic nucleus; VTA, ventral tegmental area; ZI, zona incerta; V4, fourth ventricle. Reproduced from Salinas et al. (200). Creative common license available at: http://creativecommons.org/licenses/by/4.0/.

Updated technology, which provides a fully automated analysis via integration with the Allen Brain Atlas (AIBS), has enabled a more unbiased approach and the quantitative description of the number of liraglutide uptake sites in rodent brain, as shown in Figure 9 (200). New methods also involve a fully automated reading of cFOS activation following whole brain staining. Brain uptake and activation (cFOS) can then be digitally analyzed, and compared with other studies in the AIBS. With this analysis it appears plausible that hindbrain activation—via the AP and, potentially, the NTS—is communicated to the lateral parabrachial nucleus (lPBN), which then activates further neuronal populations (e.g., in the amygdala). This is in full agreement with the finding that GLUT neurons are essential for the effect of liraglutide. The neurons in the lPBN are important for several feeding circuits (201), and may be best described as an important relay station (200).

Some of the GLP-1R populations in the brain are involved in reward-related feeding behavior. GLP-1/GLP-1RAs have been shown to affect both basal and reward-related feeding, and increase dopamine transporter levels (202, 203). A study in rats showed that liraglutide was associated with a significant reduction in total calorie intake as well as changes in food preference, including a significant decrease in candy consumption (vs. diabetes-induced obesity [DIO] controls and vildagliptin) and an increase in chow consumption (vs. DIO controls) (204). While such a study setup is difficult to replicate in humans, a study with semaglutide in patients with obesity did indicate a change in food preference and a positive effect on reward (181).

Studies with semaglutide indicate that it has better uptake in the brain compared with liraglutide (205), but whether this completely explains the difference in weight-loss effects has not been established. What is established is that the reduction in energy intake observed with semaglutide is approximately twice that with liraglutide (180, 181).

It is worth mentioning that the role of GLP-1 as a neuropeptide may be broader than currently recognized, and may go beyond mediating body weight and food intake lowering. GLP-1Rs in the brain have been described as being involved in, but not essential for, memory and learning, and depression and anxiety (119). Publications report a potentially important role in diseases such as Alzheimer's, where liraglutide has been shown to prevent tau tangles in a mouse model (206). A small clinical study showed an increase glucose uptake in the brain but did not show an effect of liraglutide on cognition (207). A larger study is ongoing (208). While no studies have indicated a beneficial effect of liraglutide or semaglutide in Parkinson's Disease, a study with exenatide has indicated that long-acting GLP-1RAs may prevent deterioration in motor symptoms in patients with the condition (209), and the suggested mechanism may be a microglia-initiated effect to prevent astrocytes from forming a toxic phenotype (121, 210).

### CV Risk Reduction

GLP-1 has several effects, other than glucose and body weight reduction, that may impact CV outcomes beneficially. Effects documented clinically include decreased BP, reduced postprandial lipid levels [triglycerides (TGs), apolipoprotein B48 (ApoB48)] and a smaller effect on fasting lipids and reduced inflammation (76).

The first animal studies showing that GLP-1 potentially had effects on the CV system was published in 2002, and the first study to show cardioprotection was in 2008 (102, 211), but it was not until the LEADER cardiovascular outcomes trial (CVOT) with liraglutide, published in 2016, that a reduction in CV risk with a GLP-1RA was documented (100).

LEADER was a post-approval trial mandated by the FDA as part of the changed guidelines (212), following the CV safety issue associated with the peroxisome proliferator-activated receptor (PPAR) class of agents. In the LEADER trial, a total of 9,340 subjects with T2D and high CV risk were randomized to liraglutide or placebo and followed-up for a median of 3.8 years (100). LEADER showed a 13% reduction in the primary outcome of major adverse cardiovascular events (MACE) with liraglutide vs. placebo (hazard ratio [HR], 0.87; 95% confidence intervals [CI], 0.78 to 0.97; *p* < 0.001 for non-inferiority, [margin of 1.3 for upper boundary of 95% CI of the HR], *p* = 0.01 for superiority), with a numeric effect on all three individual MACE components (CV death, non-fatal stroke and non-fatal myocardial infarction [MI]) (100).

While few biomarkers were included in the LEADER trial and no formal mediation analyses have been conducted that can firmly establish a mechanism, the consistent effect of liraglutide on all three MACE components led to the hypothesis that it has an impact on underlying atherosclerosis. The effects on HbA_1c_, body weight and systolic BP seem too small for them to be important mediators; at 36 months the mean difference in these parameters with liraglutide vs. placebo was −0.4% (95% CI, −0.45 to −0.34), −2.3 kg (95% CI 2.5 to 2.0) and −1.2 mmHg (95% CI 1.9 to 0.5), respectively (100). One *post-hoc* analysis of the LEADER data showed that subjects with a lower number of hypoglycemic events had lower CV risk, but the cardioprotective effects of liraglutide appeared to be largely independent of reductions in hypoglycemia (213). Another suggested a lower risk of diabetic foot ulcer-related amputations with liraglutide compared with placebo (214). A third *post-hoc* analysis did not find evidence for a reduced risk in CV outcomes following a MI (215, 216). Lastly, a *post-hoc* analysis showed a reduction in major CV outcomes in both subjects with polyvascular and single vascular atherosclerotic disease (216). Overall, it seems plausible that an effect on atherosclerosis could be involved in the CV risk reduction observed with liraglutide. This would be in contrast to the findings from the EMPA-REG OUTCOME trial, where the positive effect of the sodium-glucose cotransporter-2 inhibitor empagliflozin appears to be primarily driven by an effect on CV death, which a mediation analysis showed to be partially due to a hemodynamic effect (217).

The SUSTAIN 6 CVOT with semaglutide (0.5 and 1.0 mg) had a similar setup to LEADER, except it was conducted pre-approval, involved fewer subjects (~3,300 with established CV disease, chronic kidney disease or CV risk factors) and had a shorter duration (median observation time 2.1 years) (218). There was a 26% reduction in the risk of MACE (first occurrence of cardiovascular death, non-fatal MI, or non-fatal stroke) with semaglutide vs. placebo (HR 0.74; 95% CI, 0.58 to 0.95; *p* < 0.001 for non-inferiority [margin of 1.8 for upper boundary of 95% CI of the HR]), driven by reductions in the risk of MI and stroke (218). The trial demonstrated substantial reductions in HbA_1c_ (0.5 mg, −1.1%; 1.0 mg, −1.4%) and body weight (0.5 mg, −3.6 kg; 1.0 mg, −4.9 kg) with semaglutide vs. placebo. These findings have also been proposed to be consistent with an effect on atherosclerosis.

Two other CVOTs with GLP-1RAs, lixisenatide OD and exenatide OW, failed to show a risk reduction (RR) for the combined MACE endpoint (219, 220). Lixisenatide is a short-acting GLP-1RA dosed OD, whereas exenatide is a short-acting GLP-1 that has been modified in a sustained release formulation to make OW dosing possible. A hypothesis relating to GLP-1RA-induced CV RR may be that all long-acting GLP-1RAs will have some level of CV RR, although the specific impact of each compound may differ. While differences may be linked to the varying effect of each compound on HbA_1c_ (semaglutide>liraglutide>exenatide>lixisenatide) and body weight (semaglutide>>liraglutide>exenatide>lixisenatide), a recent CVOT with albiglutide showed that long-acting GLP-1RAs may reduce CV risk independently, at least in terms of their effect on weight loss (221). In that study, while albiglutide had limited effects on blood glucose and almost no effect on body weight, greater CV RR was confirmed with albiglutide vs. placebo.

The available scientific literature on the effect of GLP-1, liraglutide and semaglutide on other factors that may impact CV outcomes is outlined below, with focus on studies from our laboratories.

#### Effects on the Heart

In 2009, a study with liraglutide showed improved cardiac output and protection against ischemic injury in a mouse model with 28 days follow-up (222). While a similar study in pigs did not confirm these findings, the follow-up period was only 3 days, which may have been insufficient time to reveal a positive effect (223). Initial studies suggested GLP-1R expression on cardiomyocytes (102), but this was subsequently shown to be incorrect (103). GLP-1Rs are expressed in the heart, but it is unclear if they have a role in cardioprotection, or if they are primarily involved in the regulation of BP and heart rate (103, 104, 224). A randomized, controlled clinical trial with liraglutide showed a 38% decrease (from a baseline of 40.6 ± 38.6 ng/l) in brain natriuretic peptide, a marker of left ventricular dysfunction (225).

#### Postprandial Lipids

GLP-1RAs do not have specific effects on fasting lipids, other than those expected as a consequence of their weight-loss effects, but they do appear to positively affect postprandial lipid levels. A publication in 2006 showed acute GLP-1 infusion markedly lowered postprandial TGs (226). The effect was initially thought to be secondary to reduced gastric emptying, however, it was later shown to be an independent effect (227, 228). This effect has now been well-documented for both liraglutide and semaglutide, with randomized, placebo-controlled trials demonstrating a lowering of both TGs and ApoB48 following a fat-enriched breakfast (191, 228). TGs were 28% (AUC_0−8h_) and 57% (iAUC_0−8h_) lower with liraglutide vs. placebo, and ApoB48 was 33% (AUC_0−8h_) and 57% (iAUC_0−8h_) lower (228). With semaglutide postprandial (iAUC_0−8h_) TG and ApoB48 levels were 40.7 and 49.6% lower compared with placebo (191). The studies differed in regard to the fat content of the meal: in the semaglutide study it was lower. In both studies, the postprandial lipid profiles were assessed at steady state, when little effect remains on gastric emptying (191, 228). The first study to describe a potential mechanism for this effect on postprandial lipids was published in 2010 (229). The study, in hamsters and rats, suggested that GLP-1 may decrease intestinal lipoprotein biosynthesis and secretion (229). An uncontrolled kinetic, clinical study with liraglutide showed a reduction in ApoB48 production and an increase in ApoB48 catabolism (230). This effect on postprandial lipids is potentially interesting in relation to CV RR, as TGs and remnant cholesterol are emerging as independent risk factors for CV disease (231–234).

#### Atherosclerosis

An uncontrolled clinical trial has suggested that liraglutide can reduce atherosclerotic burden in humans; in this study there was a significant reduction in carotid intima-media thickness during an 18-month follow-up (235). As no randomized, controlled trials have directly investigated atherosclerosis, studies in animals are currently the main source of further information. The first study with liraglutide was published in 2013 (236). In this study, using pro-atherogenic ApoE-deficient mice, liraglutide attenuated the development of atherosclerosis and improved plaque stability, as measured by a change in plaque composition, specifically an increase in collagen content and alfa-smooth muscle action. There was a reduction in plaque size with liraglutide, and this was found to be GLP-1R-dependent. No significant effect on progression of late-onset, high-burden atherosclerotic disease was observed, and there was no significant endothelial cell dysfunction in this animal model. This publication suggested a potential role for liraglutide in the prevention and stabilization of atherosclerotic vascular disease, together with possible protection against major CV events.

Other studies have, subsequently, also shown an effect of liraglutide in atherosclerosis. In one such study, treatment with liraglutide suppressed foam cell formation through a reduced uptake of oxidized low-density lipoprotein (LDL) cholesterol, potentially caused by a downregulation of the scavenger receptor CD36 (237).

**Figure 10:**
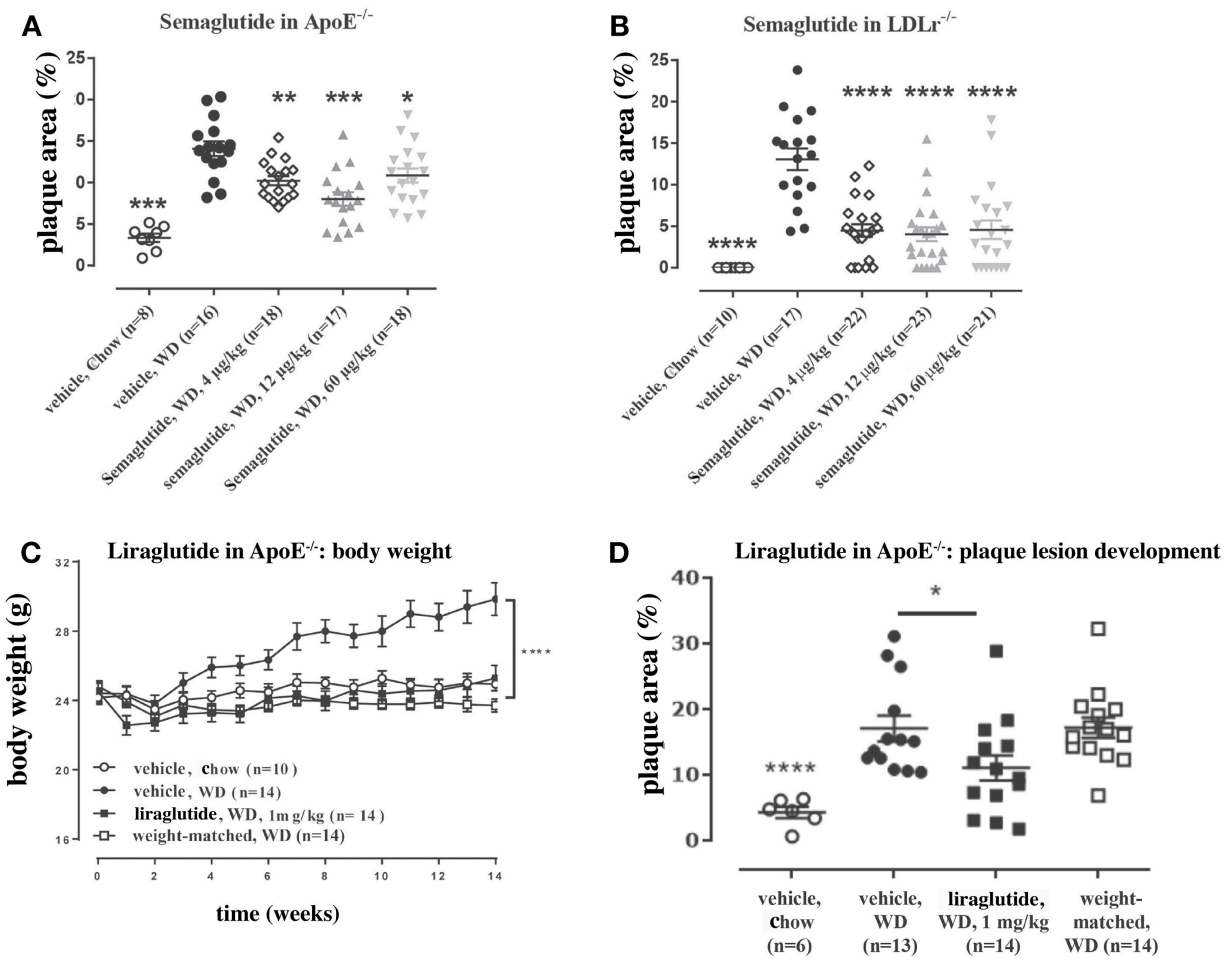
Plaque lesion development with semaglutide and liraglutide in apolipoprotein E and low-density lipoprotein receptor knockout mice aorta, and effect on atherosclerosis. **(A)** Semaglutide significantly decreased WD-induced plaque lesion development at all dose levels in ApoE^−/−^ mice: ^*^p = 0.0266, ^**^p = 0.0046, ^****^p < 0.0001 vs. vehicle WD, and **(B)** LDLr^−/−^ mice: ^****^p < 0.0001 vs. vehicle WD. **(C)** WD-induced increases in body weight were significantly lowered by liraglutide and in the weight-matched comparator: ^****^p < 0.0001 vs. vehicle, WD. **(D)** Liraglutide significantly attenuated WD-induced plaque lesion development, whereas the weight-matched comparator group did not: ^*^p = 0.0448, ^****^p < 0.0001 vs. vehicle, WD; liraglutide, WD vs. weight-matched, WD, *p* = 0.06). ApoE, apolipoprotein E; LDLr, low-density lipoprotein receptor; WD, Western diet. Reprinted from Rakipovski et al. (111), Copyright 2018, with permission from Elsevier.

We conducted studies that assessed the effects of liraglutide and semaglutide in LDL receptor and ApoE knockout mice. The key results are outlined in Figure 10 and described below. Both compounds attenuated aortic atherosclerotic plaque lesion size independent of their effects on: diabetes (as the animal models were non-diabetic); body weight (as similar effects were observed for doses that affected body weight or not, and control experiments with weight matching did not lead to plaque size effects); and lipids (as only high doses affected plasma TGs, whereas low doses also affected plaque size) (111).

#### Inflammation

**Figure 11:**
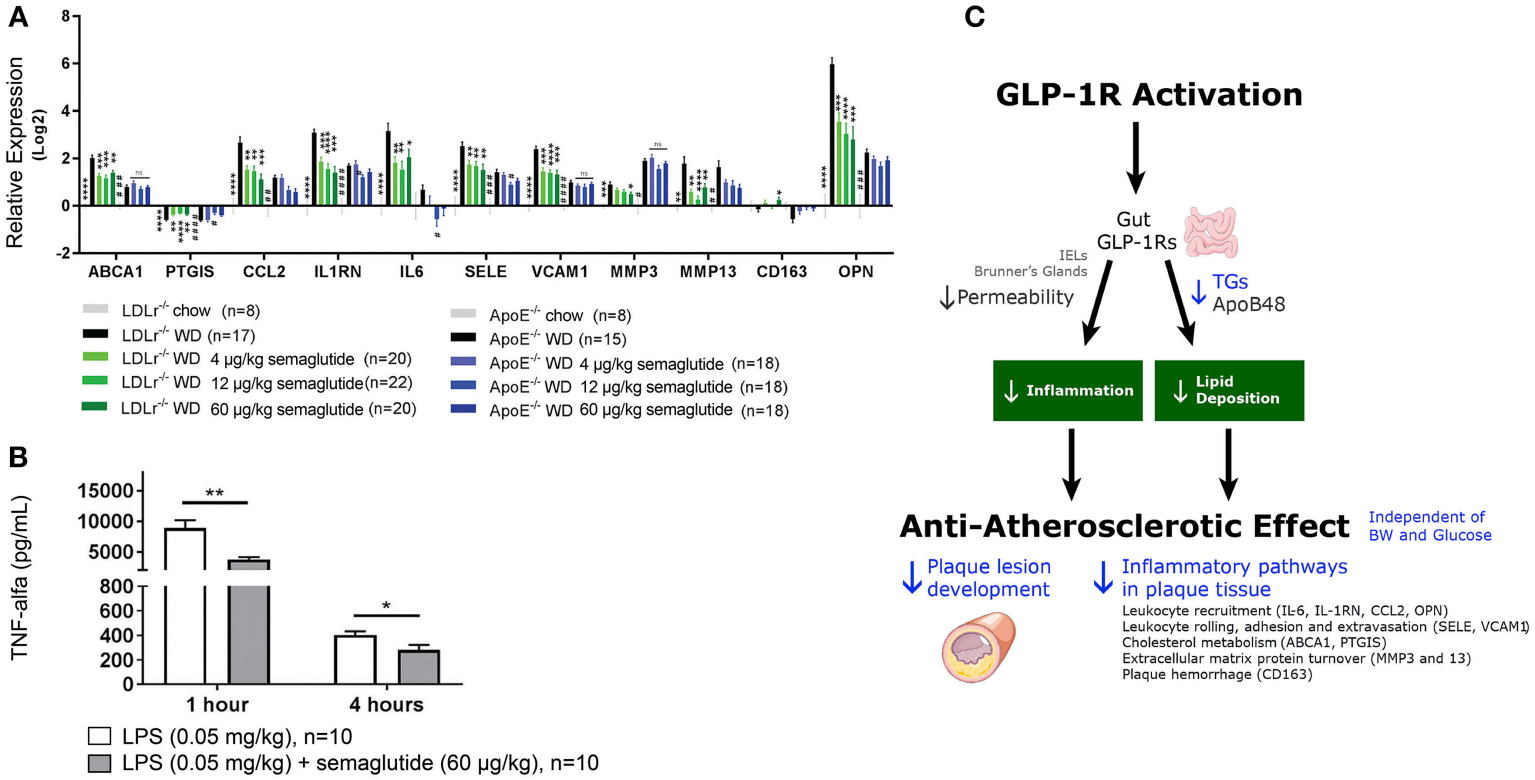
Effects of GLP-1RAs on atherosclerosis is hypothesized to occur via inflammation. **(A)** Gene expression changes by WD and semaglutide (compared with vehicle-dosed chow-fed animals), exemplifying genes that represent pathways with well-described relevance to plaque formation and the pathophysiology of atherosclerosis, Benjamini–Hochberg corrected *p*-values; ^****^p < 0.0001; ^***^p < 0.001; ^**^p < 0.01; ^*^p < 0.05 vs. LDLr^−/−^, WD; ^*####*^*p* < 0.0001, ^*###*^*p* < 0.001; ^*##*^*p* < 0.01; ^#^*p* < 0.05 vs. ApoE^−/−^, WD. **(B)** Subcutaneous administration of semaglutide in an LPS inflammation model reduced plasma levels of TNF-alfa, ^**^p = 0.0024 vs. vehicle at 1 h and *p* = 0.048 vs. vehicle at 4 h. **(C)** Proposed model illustrating how long-acting GLP-1RAs could reduce atherosclerotic burden. ABCA 1, ATP-binding cassette transporter 1; ApoE, apolipoprotein E; BW, body weight; CCL2, chemokine [C-C motif] ligand 2; CD, cluster of differentiation; GLP-1RA, glucagon-like peptide-1 receptor agonist; IEL, intestinal intraepithelial lymphocyte; IL, interleukin; LDL, low-density lipoprotein; LPS, lipopolysaccharide; MMP, matrix metalloproteinase; OPN, osteopontin; PTGIS, prostaglandin I2 synthase; SELE, selectin E; TG, triglyceride; TNF-alfa, tumor necrosis factor-alfa; VCAM 1, vascular adhesion molecule 1; WD, Western diet. Reprinted from Rakipovski et al. (111), Copyright 2018, with permission from Elsevier.

To further understand the mechanism by which GLP-1RAs affect atherosclerosis, the transcriptomes in aorta tissue samples were analyzed. These results are outlined in Figure 11. A number of genes related to inflammation were positively affected by semaglutide. Semaglutide partially prevented Western diet-induced changes for transcripts associated with pathways relevant to the pathogenesis of atherosclerosis, and the changes appeared to be independent of the doses used, whereas a typical dose-response relationship was seen for weight loss and TG lowering.

Inflammation is an established risk factor for CV disease (238). While C-reactive protein (CRP) is the most established systemic inflammatory CV risk marker, recent data indicate that inflammatory markers in vascular tissue [matrix metalloproteinases (MMP), osteopontin (OPN), and cathepsin D] may be more specific measures of plaque instability (239). Elevated circulating levels of OPN, a pro-inflammatory cytokine with a role in immune cell recruitment (240), have been associated with increased CV disease risk in patients with T2D (241). The genes affected by semaglutide included markers of inflammation and plaque stability that are associated with leukocyte recruitment [e.g., interleukin-1 (IL-1) receptor antagonist, IL-6, monocyte chemoattractant protein (CCL2), OPN], leukocyte adhesion [selectin E, vascular cell adhesion molecule-1 (VCAM1)], leukocyte extravasation and plaque stability (e.g., MMP3, MMP13, CCL2), and plaque rupture and hemorrhage (CD163). Several of these markers are hypothesized to be of relevance in CV disease (IL-1 receptor antagonist, IL-1 receptor, MMP3, CD163, IL-6 and its receptor) (242–245).

Clinical studies have shown that liraglutide lowers levels of markers associated with inflammation. A randomized, placebo-controlled trial showed that liraglutide reduced CRP by 35%, from a baseline of ~3.5 mg/dl, in subjects with obesity (175). In smaller studies, without a placebo control, liraglutide lowered levels of some additional markers of inflammation [sCD163 (246), selectin E, and plasminogen activator inhibitor-1 (PAI-1) (247)], while leaving others unchanged [VCAM-1, CRP, and CCL-2 (247)]. Furthermore, several *in vitro* or *ex vivo* studies have shown that liraglutide lowers inflammation. For example, liraglutide has been shown to decrease levels of tumor necrosis factor-alfa (TNF-alfa)-induced secretion of intracellular adhesion molecule, VCAM and PAI-1 from human umbilical vein endothelial cells (248). The challenge with interpreting the findings of such studies is sometimes the lack of data convincingly showing GLP-1R expression. In this case, however, the findings were supported by a randomized, placebo-controlled study of liraglutide in subjects with diabetes who showed a clinically and statistically significant reduction in PAI-1 (−25%, from a baseline of 31.4 ± 28.4 U/ml) and a statistically significant reduction in CRP (−20%, from a baseline of 4.2 ± 4.2 mg/l), but no change in TNF-alfa or IL-6 (225).

As GLP-1R mRNA in the aortic tissue was below the level of quantification in both animal models, it seems likely that GLP-1Rs in other tissues, rather than direct action of liraglutide or semaglutide on the vascular bed, contribute to the mechanism of gene regulation.

To further understand this, we conducted studies to assess how GLP-1 may affect inflammation, focusing on the role of the GLP-1Rs in the gut. For some years it has been suggested that GLP-1 improves the gut-barrier function, and that this may be mediated via intraepithelial intestinal lymphocytes (98). We suggest that GLP-1R in the mucus-secreting Brunner's glands may also be involved. As already highlighted, these glands have high GLP-1R expression in both rodents and humans, and liraglutide has been shown to upregulate genes encoding mucins in the Brunner's glands of mice (73, 87, 249), which may reduce intestinal permeability and improve systemic inflammation (250). Figure 11 shows how semaglutide decreased measures of systemic inflammation following lipopolysaccharide challenge (111).

Although it has previously been highlighted that discrepancies may be apparent when comparing the regulation of murine and human genes involved in atherosclerosis development (251), the current study showed an overlap in the pathways identified in the transcriptomic analysis and those identified in human pathophysiology and in clinical studies with liraglutide. This overlap and the findings from the CVOTs with liraglutide and semaglutide, showing an effect consistent with reduced underlying atherosclerotic burden, suggest that the anti-inflammatory effects of liraglutide and semaglutide seen in animal models may translate to humans.

## The Technology Behind Oral Semaglutide, and the Mechanism of Its Absorption

Historically, peptides and proteins have offered efficacious treatment options either as replacements in diseases where the native peptide- or protein-based hormone is not being produced endogenously, or it is being produced or secreted in smaller amounts than required (e.g., insulin in type 1 diabetes, growth hormone in growth disorders, factor VIII in hemophilia disorders).

Insulin was discovered as a life-saving drug for the treatment of type 1 diabetes and has also had a profound impact on the treatment of T2D. Despite various delivery mechanisms being tested (e.g., pulmonary, buccal, and sublingual), low bioavailability and uncertainty in terms of safety has limited the development and, as such, almost all available insulins are injectable. In the past 15 years, GLP-1RAs have emerged as efficacious therapies in T2D and obesity, with evidence also indicating potential in CV disease and NASH. Due to the relatively larger safety window of GLP-1, compared with insulin, oral delivery of GLP-1 now appears to be feasible.

Oral delivery is known as a desirable approach in terms of patient convenience, but it has not been possible to achieve acceptable bioavailability of large hydrophilic peptides and proteins via this approach; oral absorption typically requires a low molecular weight and a certain hydrophobicity (252). One approach could be to use small-molecule GLP-1RAs. While such compounds have been described, none have yet been considered credible pharmacological candidates (253, 254) due to a combination of factors, including insufficient potency, lack of specificity of binding to the GLP-1R and suboptimal half-life.

Another approach could be to use an absorption enhancer; we have explored this approach during the past 10 years. Such an enhancer-driven approach now appears to be feasible for oral absorption of semaglutide, with phase 3 clinical trials ongoing. Orally administered semaglutide comprises a tablet formulation with a pharmaceutically inactive small-molecule enhancer SNAC (N-[8-(2-hydroxybenzoyl)amino] caprylate). Phase 2 clinical data showed that, while bioavailability was low following oral administration (~1%), it led to the same efficacy in terms of both glycemic control and weight loss as subcutaneously delivered semaglutide (255). SNAC was originally developed by Emisphere, and has been granted GRAS (generally recognized as safe) status by the FDA for use with food supplements, vitamins and dietary ingredients (256). Although SNAC has been tested in medium-scale clinical trials (e.g., with salmon calcitonin and parathyroid hormone (257, 258) and has shown some positive effects, its molecular mechanism of absorption is not fully understood (257, 259–261).

**Figure 12:**
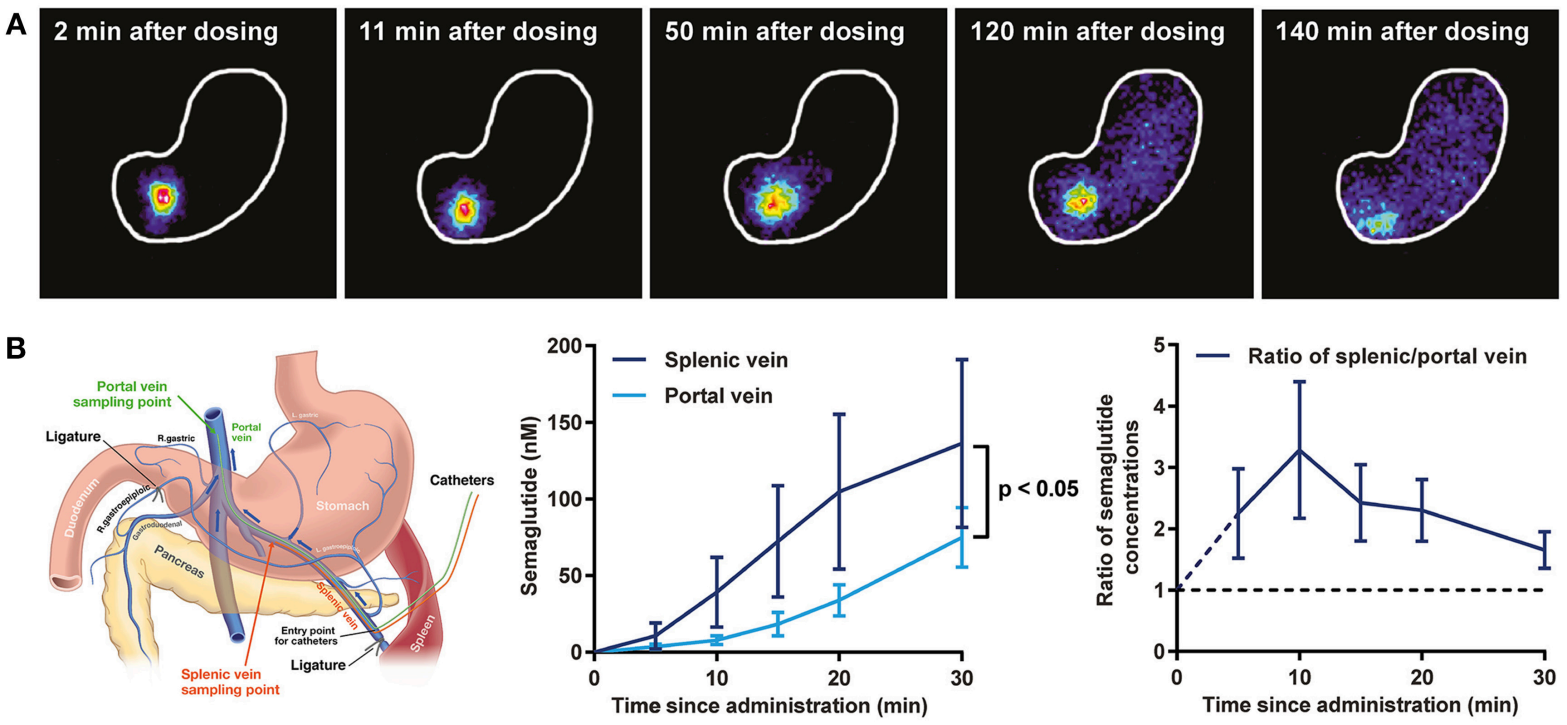
Anatomical site of absorption of oral semaglutide. **(A)** Gamma scintigraphic imaging of tablet erosion in the stomach from 2 to 140 min after a single dose of oral semaglutide (10 mg) in a representative healthy individual. White line outlines the stomach; colors within the stomach (red/yellow/green/blue) represent the tablet core and released radioactivity. **(B)** Illustration of the splenic vein, which drains the gastric cavity, and the portal vein, which drains the gastrointestinal system. Mean semaglutide plasma concentration–time profiles in the splenic and portal veins after a single dose of oral semaglutide (10 mg) in beagle dogs (*n* = 15). R. gastric, right gastric; L. gastric, left gastric; R. gastroepiploic, right gastroepiploic; L. gastroepiploic, left gastroepiploic. The ratio and 95% CI of the splenic vs. portal veins for AUC_0−30min_ were calculated [1.94 (1.15 to 2.74)], and statistical significance was determined on the basis of a null hypothesis value of 1 (*p* < 0.05). The horizontal dashed line (right) represents similar semaglutide plasma concentrations in the splenic and portal veins. Error bars show ±SEM calculated on the original scale or calculated on a log-scale and back-transformed to the original scale. From Buckley et al. (262). Reprinted with permission from the American Association for the Advancement of Science (AAAS).

Oral absorption of small molecules without enhancers mostly occurs in the intestine, and this has been assumed to be the case for peptide-based drugs with enhancers. However, peptide absorption in the intestine is a challenge, as numerous peptidases may degrade substances before they can be absorbed. One solution is to alter the peptide to protect it from enzymatic degradation. This can, however, lead to immunogenicity. An important aspect of oral semaglutide is that absorption likely takes place in the stomach. The key data that illustrate this are outlined in Figure 12 and the text below, with studies in both humans and animals (262). Human data showed that tablet dissolution occurs in the stomach, and animal (canine) data demonstrated that uptake initially occurs from the splenic, and not the portal, vein. Together these data indicate that semaglutide uptake occurs in the stomach. The paper further illustrates that uptake occurs in a localized process, where the concentration of both semaglutide and SNAC (which is also absorbed) decreases rapidly as a function of distance to the tablet's physical location in the stomach (262). Co-formulation with SNAC is required for absorption, as the absorption of other drugs administered concomitantly to (but not co-formulated with) SNAC are not affected in any clinically relevant manner. Furthermore, absorption is specific to the compounds used: for example, liraglutide is not absorbed when co-formulated with SNAC, likely because it is too hydrophobic. Similarly, the use of a close analog of SNAC does not lead to the absorption of semaglutide. Another factor that aids the process is a localized increase in pH facilitated by SNAC, which leads to better solubility of semaglutide, as well as protection against degradation by stomach peptidases that have little activity at a neutral pH.

**Figure 13:**
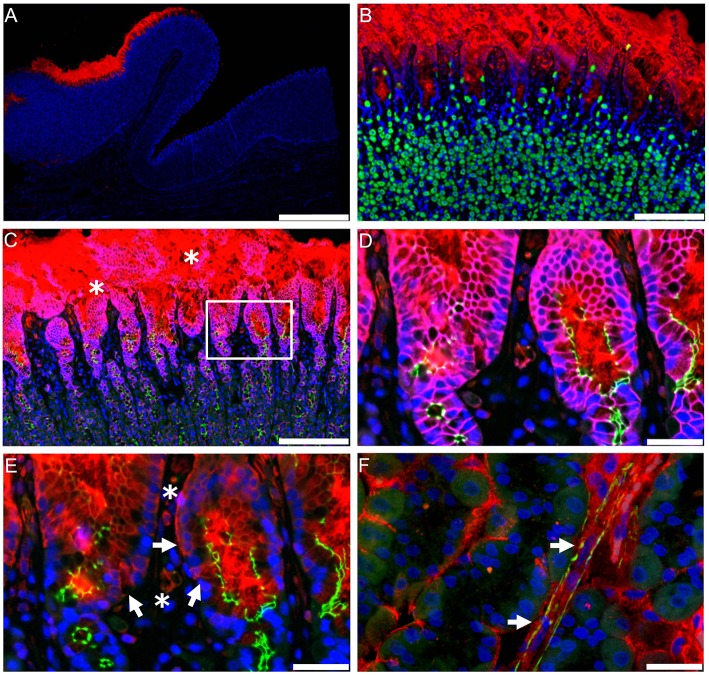
Absorption and localization of semaglutide in dog gastric mucosa. **(A)** Local 3F15 (semaglutide) immunofluorescence reactivity (red) under the tablet. Nuclei counterstained with 4′,6-diamidino-2-phenylindole (DAPI; blue). Scale bar, 2 mm. **(B)** 3F15 reactivity (red) restricted to the neck region. The bulk of H_+_/K_+_ ATPase (green)–positive parietal cells reside in deeper layers, but a few scattered parietal cells can be found in the neck region exposed to luminal semaglutide. Scale bar, 200 μm. **(C)** 3F15 (red), β-catenin (purple), ZO1 (green), and DAPI (blue). Sloughing of the uppermost region of the epithelium is marked by white asterisks; semaglutide is also detected in deeper, intact layers (white box). Scale bar, 200 μm. **(D)** Higher magnification of the boxed area in **(C)**. Intact tight junctions labeled with apical ZO1 (green) in direct contact with luminal semaglutide. Scale bar, 40 μm. **(E)** Same region as **(D)** without β-catenin. Intracellular 3F15 reactivity (red) is observed in mucosal cells (marked by white arrows). 3F15 also detects semaglutide in capillaries under the epithelium (marked by white asterisks). Scale bar, 40 μm. **(F)** Maximum projection image from a 63 × confocal 11-μm z stack, showing semaglutide (red) associated with a blood vessel (marked by white arrows) labeled with smooth muscle actin (green). Scale bar, 40 μm. From Buckley et al. (262). Reprinted with permission from the American Association for the Advancement of Science (AAAS).

Oral absorption principally occurs via two mechanisms: transcellular and paracellular. The absorption of semaglutide and SNAC is primarily transcellular, as shown *in vitro* using gastric mucosa in a chamber setup, a gastric cell line to document intracellular uptake and EDTA as a control enhancer facilitating paracellular uptake. Figure 13 shows data related to the molecular mechanism of absorption. Transcellular uptake of semaglutide was demonstrated *in vivo* in dog gastric mucosa and confirmed at the electron micrograph level in rats. The molecular mechanism for transcellular uptake appears to involve SNAC partitioning into the cell membrane, due to its lipophilic nature, and affecting membrane fluidity. This results in the rapid absorption of both semaglutide and SNAC. The effect is transient, and appears not to cause damage, probably because cell turnover in the gastric mucosa is rapid—as observed following ethanol absorption in the stomach (263–265).

In conclusion, through a variety of tests (cell lines, tissue cultures, *in vivo* animal models, IHC including at the electron micrograph level, imaging) and clinical trials (scintigraphy imaging, drug–drug interactions), oral semaglutide co-formulated with SNAC has been shown to be absorbed in the stomach in a localized, transient, transcellular process, where a local increase in pH provides solubility and protection against enzymatic degradation.

## Summary

The development of the long-acting GLP-1 analogs liraglutide and semaglutide, using albumin binding to extend the half-life, represents a significant advance in GLP-1-based therapies. The improvement in glycemic control and reduction in body weight with these compounds offers benefits to patients with T2D, with liraglutide (3.0 mg) also providing a treatment option for those with obesity and semaglutide under investigation for this indication. Furthermore, both liraglutide and semaglutide have been shown to reduce CV risk in patients with T2D, a finding that has not been observed with short-acting GLP-1RAs. Given that CV complications remain a leading cause of morbidity and mortality in people with T2D (266), despite substantial efforts to reduce risk using CV medications, the availability of antidiabetes medications that can contribute to risk reduction is a significant advance.

We await the results of the phase 3 trials with the oral formulation of semaglutide. Beyond diabetes, GLP-1RAs are also being investigated in conditions such as NASH. Thus, it seems likely that we can look forward to further progress in the field of GLP-1-based therapy.

## Author Contributions

JL provided a full final draft of the section on the design of liraglutide and semaglutide. LK provided a full final draft of the rest of the article, a selection of figures.

### Conflict of Interest Statement

LK and JL are full-time employees and shareholders of Novo Nordisk. LK is a named inventor of liraglutide, and JL of semaglutide; the IP is fully owned by Novo Nordisk, who markets both compounds for the treatment for obesity and/or diabetes.
